# Circulating Tumor Cell Transcriptomics as Biopsy Surrogates in Metastatic Breast Cancer

**DOI:** 10.1245/s10434-021-11135-2

**Published:** 2022-01-09

**Authors:** Alexander Ring, Daniel Campo, Tania B. Porras, Pushpinder Kaur, Victoria A. Forte, Debu Tripathy, Janice Lu, Irene Kang, Michael F. Press, Young Ju Jeong, Anson Snow, Yue Zhu, Gabriel Zada, Naveed Wagle, Julie E. Lang

**Affiliations:** 1 Division of Surgical Oncology, Department of Surgery and University of Southern California Norris Cancer Center, University of Southern California, Los Angeles, CA USA; 2 Present Address: Department of Hematology and Medical Oncology, University Hospital Zurich, Zurich, Switzerland; 3 Department of Biological Sciences, University of Southern California, Los Angeles, CA USA; 4 Division of Medical Oncology, Department of Medicine, SUNY Downstate Medical Center, New York, NY USA; 5 Department of Breast Medical Oncology, UT MD Anderson Cancer Center, Houston, TX USA; 6 Division of Medical Oncology, Department of Medicine and University of Southern California Norris Cancer Center, University of Southern California, Los Angeles, CA USA; 7 Department of Pathology and University of Southern California Norris Cancer Center, University of Southern California, Los Angeles, CA USA; 8 Department of Surgery, Catholic University of Daegu School of Medicine, Daegu, Republic of Korea; 9 Department of Neurosurgery and University of Southern California Norris Cancer Center, University of Southern California, Los Angeles, CA USA; 10 Present Address: Division of Breast Services, Department of General Surgery, Cleveland Clinic Breast Cancer Program, Cleveland, Ohio USA

## Abstract

**Background:**

Metastatic breast cancer (MBC) and the circulating tumor cells (CTCs) leading to macrometastases are inherently different than primary breast cancer. We evaluated whether whole transcriptome RNA-Seq of CTCs isolated via an epitope-independent approach may serve as a surrogate for biopsies of macrometastases.

**Methods:**

We performed RNA-Seq on fresh metastatic tumor biopsies, CTCs, and peripheral blood (PB) from 19 newly diagnosed MBC patients. CTCs were harvested using the ANGLE Parsortix microfluidics system to isolate cells based on size and deformability, independent of a priori knowledge of cell surface marker expression.

**Results:**

Gene expression separated CTCs, metastatic biopsies, and PB into distinct groups despite heterogeneity between patients and sample types. CTCs showed higher expression of immune oncology targets compared with corresponding metastases and PB. Predictive biomarker (*n* = 64) expression was highly concordant for CTCs and metastases. Repeat observation data post-treatment demonstrated changes in the activation of different biological pathways. Somatic single nucleotide variant analysis showed increasing mutational complexity over time.

**Conclusion:**

We demonstrate that RNA-Seq of CTCs could serve as a surrogate biomarker for breast cancer macrometastasis and yield clinically relevant insights into disease biology and clinically actionable targets.

**Supplementary Information:**

The online version contains supplementary material available at 10.1245/s10434-021-11135-2.

Metastatic breast cancer (MBC) is responsible for virtually all BC deaths. MBCs are often discordant in biomarker profiles when compared with the primary tumor.^1^ The American Society of Clinical Oncology guidelines call for biopsies of metastases for biomarker testing to guide decision making for systemic therapy.^2,3^ However, not all metastatic sites are amenable to safe percutaneous biopsy. Improved survival in MBC is in large part due to the availability of targeted therapies.^4,5^ Most patients with MBC unfortunately develop treatment resistance.^6^

Although molecular profiling of tumors may predict targeted therapy opportunities, primary tumor or single metastatic biopsy site driven approaches may not represent multiple non-overlapping oncogenic alterations driving biology in patients with multiple metastatic sites.^7^

Circulating tumor cells (CTCs) hold significant potential as liquid biopsies obtained via minimally invasive blood draws for the real-time assessment of a patient’s tumor biology and heterogeneity.^8,9^ CTCs have been shown to be prognostic in MBC and are present in 52–71% of MBC patients,^10^ but have not provided predictive insights for targeted therapy. Hence, CTCs have not been used extensively to guide therapy decisions. A potential issue is the selection of cell populations based on cell surface marker expression, such as the only FDA approved method via the cell search system.^11^ Another issue is that mere enumeration of CTCs or technological limitations have hampered the capability to interrogate CTC biology and gain insights into potentially targetable lesions.^8^

Many sequencing approaches available now, including those with clinical application, focus on DNA sequencing, but there are major concerns that not all DNA mutations are expressed.^12^ Refinements in RNA-Seq technology now enable detailed molecular profiling of CTCs^13^ beyond gene expression, offering the potential for predicting treatment options via a liquid biopsy.

We hypothesized that molecular characterization via whole transcriptome RNA sequencing of CTCs isolated in an unbiased, marker independent fashion can capture disease heterogeneity of MBC and may serve as a surrogate for the analysis of macrometastases to identify predictive biomarkers, potentially leading to new target discovery and explaining treatment resistance.

## Materials and Methods

### Study Design and Patient Population

The project was designed as an observational study to evaluate whether RNA-sequencing (RNA-Seq) of CTCs can identify potential treatment targets. A total of 21 treatment naïve female MBC patients were prospectively enrolled at the Keck Medical Center and Norris Comprehensive Cancer Center at the University of Southern California (USC). Each patient underwent biopsies of macrometastases for clinical diagnostic purposes collected at baseline (prior to therapy for MBC) or upon disease progression prior to switching therapy. A baseline PB draw of 7.5 ml in an EDTA tube for CTC RNA-Seq was required for inclusion. Data from 19 patient samples passing quality criteria were included in further analysis. Four of the 19 patients with progressive disease returned for repeat PB draws after approximately 6 months of treatment to track the changes in CTC biology over time. Response to therapy was assessed based on RECIST criteria.^14^ All procedures, including written patient informed consent, were approved by the Institutional Review Board (IRB HS-14-00595 and HS-11-00208) at USC. This study was compliant with the REMARK criteria.^15^

## Molecular Marker-Independent CTC Isolation

The Parsortix microfluidics filtration system (ANGLE plc, Surrey, United Kingdom) efficiently captures and highly enriches CTCs in a cell surface marker independent manner based on size and deformability,^16–19^ reducing the number of contaminating white blood cells (WBCs) by roughly 5 orders of magnitude. The device has a Diagnostic Devices Directive CE Mark for clinical use in Europe. We have previously validated the capture efficiency of the device in our lab using breast cancer cells spiked into peripheral blood samples (Supplementary Fig. S1). A capture cassette with a critical gap of 10 microns was used to enriched CTCs.^19^ Cell pellets were resuspended in 10 µl of lysis buffer (NuGEN Technologies, Inc., San Carlos, CA) and stored at −80 °C for further use. Rigorous device cleaning was performed between samples. This cell surface marker independent approach allowed for the capture of heterogeneous CTC populations, including EpCAM negative cells and clusters of CTCs.^17^ As processing time is critical to maximize capture efficiency^20^ total time from blood draw to CTC harvest did not exceed 2 h. As negative controls, phosphate buffered saline (PBS) samples and PB from 5 healthy female donors were processed.

### Sample Preparation and Whole Transcriptome RNA-Seq and Sanger Sequencing

Either 50 ng of RNA from a metastasis or PB, isolated with a TRIzol or RiboPure kit (both Thermo Fischer Scientific, Waltham, MA), respectively, or 2 μl of CTC lysate, were used to create cDNA for sequencing library preparation using the Ovation RNA-Seq System V2 and Ovation Ultralow Library System V2 (NuGEN Technologies, San Francisco, CA). Details regarding isolation and preparation of RNA can be found in Supplementary File S1. Sequencing was done on an Illumina HiSeq 2500 (Illumina, San Diego, CA) performing 100 base pair paired-end RNA-Seq using five samples per lane. Sanger sequencing was performed by Genewiz (South Plainfield, NJ, USA) and the sequencing data was analyzed manually with 4Peaks (Nucleobytes, Aalsmeer, Netherlands) (Supplementary File S1, Supplementary Table S1). RNA-Seq data quality control and mapping were performed as previously described^21^ (Supplementary File S1). For somatic SNV (single nucleotide variant) calling, the FASTQ files were processed following the Best Practices Workflow for variant calling with RNA-Seq from the Broad Institute (Supplementary File S1). The COSMIC database and 184 known driver genes in BC from the Integrative Onco Genomics database (http://www.intogen.org/mutations/)^22^ were investigated for known SNVs in our data set. The driver gene analysis was done using Maftools.^23^ We curated a list of 64 BC related genes with clinical and preclinical therapeutic, prognostic, or diagnostic implications, performing an extensive literature search^24^ (Supplementary Table S2) representing breast cancer relevant pathways (EGFR/RAF/MEK, IGF-1/PI3K/AKT/mTOR, WNT/NOTCH/Hedgehog/FGF/MET, DNA damage repair, cell cycle, hormone receptor signaling, tumor suppressors, and tumor immunology). The FASTQ files, as well as the corresponding read count files for each sample were deposited in the Gene Expression Omnibus database (GSE113890).

### Statistical Analysis

Statistical analyses were conducted using GraphPad Prism (San Diego, CA, USA). For differences in gene expression, two-way ANOVA was used. For SNV comparison, the Wilcoxon sign-rank test and Friedman test were used. The number of uniquely mapped reads was compared using Kruskal-Wallis and Dunn’s multiple comparison tests.

## Results

### Patient Characteristic and Grouped Gene Expression Analysis Separates Sample Type

Table 1 lists all 19 patients with clinical annotations, including site of metastasis, biomarker [ER, progesterone receptor (PR), and HER2] expression as well as treatments received (two patient samples were excluded due to low read counts). The number of uniquely mapped reads was comparable between all sample groups (median uniquely mapped reads: CTCs 30,962,730 ± 19,535,767; metastases 40,580,689 ± 14,659,648; PB 43,727,727 ± 22,600,318; CTCs vs PB *p* = 0.094; CTCs vs metastases *p* = 0.094; PB vs metastases *p* > 0.99. Median coverage: CTCs 104X ± 64; metastasis 139X ± 64; PB 153X ± 93; CTCs vs PB *p* = 0.058; CTCs vs metastasis *p* = 0.25; PB vs metastasis *p* = 0.78) (Supplementary Table S3). We obtained an average coverage higher than 50X for all but five of the samples, and coverage greater than 100X in 86% of the samples. The negative controls (PBS samples processed by Parsortix) yielded virtually no read counts (Supplementary Table S3).

**Table 1:** Clinical annotations of all patients

Patient	Primary tumor				Metastatic site						CTC collection	
	Histology	ER	PR	HER2	Type	ER	PR	HER2	Overall treatment history	Therapy at time of metastasis diagnosis	Therapy at visit 1	Therapy at visit 2
1	N/A	Y	N	N	Pleural effusion	Y	N	N	Everolimus/exemestane, paclitaxel/capecitabine/zoledronic acid, doxorubicin, gemcitabine	Doxorubicin	Doxorubicin	Gemcitabine
2	N/A	N/A	N/A	N/A	LN	Y	Y	N	ALND + XRT, tamoxifen, anastrozole/ lanreotide	None	None	
3	IDC	N	N	N	Brain	N	N	N	Segmental mastectomy/ SLN+ IORT, docetaxel/ cyclophosphamide, XRT, capecitabine/ denosumab	Capecitabine	Capecitabine	
4	PLC	Y	Y	N	Ascites	N/A	N/A	N/A	Anastrozole/palbociclib, paclitaxel, doxorubicin	Doxorubicin	Doxorubicin	
5	N/A	Y	Y	N	Liver	Y	Y	N	Chemotherapy (unknown)/ XRT, tamoxifen, paclitaxel/cisplatin, anastrozole, palbociclib/letrozole	Letrozole + palbociclib	Letrozole + palbociclib	
6	N/A	Y	N	N	Brain	Y	N	N	Chemotherapy (unknown) + right mastectomy, anastrozole	Anastrozole	Anastrozole	
7	IDC	Y	Y	N	Breast	Y	Y	N	Lumpectomy	None	None	
8	N/A	Y	Y	N	Ascites	N/A	N/A	N/A	Lumpectomy/ chemotherapy (unknown)/ radiation, carboplatin/paclitaxel, anastrozole, fulvestrant/ palbociclib, capecitabine, doxorubicin, everolimus/exemestane	Doxorubicin	Doxorubicin + gemcitabine	Gemcitabine + exemestane + everolimus
9	IDC	Y	Y	N	Pleural effusion	Y	N	N	Dose dense adriamycin/ cyclophosphamide, MRM, paclitaxel, tamoxifen/ XRT, anastrozole, leuprolide/ zoledronic acid, fulvestrant/ leuprolide depot, capecitabine, everolimus/ exemestane, paclitaxel, doxorubicin	Paclitaxel > doxorubicin	Paclitaxel > doxorubicin	
10	IDC	Y	Y	N	Breast	Y	Y	N	Doxorubicin/ cyclophosphamide/ paclitaxel, left segmental mastectomy with lymphadenectomy, tamoxifen/ leuprolide/ zoledronic acid + XRT, exemestane/ leuprolide/ zoledronic acid, fulvestrant/ palbociclib, capecitabine + XRT,	Capecitabine	Capecitabine+ XRT	Paclitaxel
11	IDC	N	N	N	CSF	N	N	N	Adriamycin/cyclophosphamide, paclitaxel, right MRM + ALND + XRT, right craniotomy w/ removal right occipital mass + cytarabine, carboplatin/ capecitabine	Carboplatin + capecitabine	Carboplatin + capecitabine	
12	ILC	N	N	N	Bone	N/A	N/A	N/A	Segmental mastectomy, brachytherapy, docetaxel/ cyclophosphamide, doxorubicin/cyclophosphamide/ paclitaxel + XRT, anastrozole, letrozole, capecitabine	Capecitabine, letrozole	Capecitabine + letrozole	
13	IDC+IBC	N	N	N	Brain	N	N	Y	Simple mastectomy, carboplatin/taxotere+ radical mastectomy, gemcitabine/trastuzumab, surgical excision by suboccipital craniotomy + gamma knife	None	None	
14	IDC	Y	Y	N	Breast	Y	N	N	Adriamycin/ cyclophosphamide, paclitaxel, tamoxifen, mastectomy + XRT, leuprolide/ anastrozole	Leuprolide + anastrozole	Leuprolide + anastrozole	
15	IDC	Y	Y	N	Pleural effusion	Y	N	N	Adriamycin/ cyclophosphamide /docetaxel, BMX + bilateral ALND, capecitabine + XRT, tamoxifen, leuprolide/ zoledronic acid, capecitabine, gemcitabine/ carboplatin	Capecitabine	Capecitabine	Gemcitabine + carboplatin
16	IDC	N/A	N/A	N/A	Pleural effusion	N	N	N	Adriamycin/ cyclophosphamide, paclitaxel, tamoxifen, mastectomy	None	None	
17	N/A	Y	N	N	CSF	Y	N	N	Mastectomy/ adjuvant chemotherapy/ XRT + adjuvant hormonal therapy, anastrozole, fulvestrant, paclitaxel/ capecitabine, carboplatin, vinorelbine	Gemcitabine	Vinorelbine	
18	IDC+ILC	Y	N	N	Skin	Y	N	N	Letrozole + XRT	XRT + letrozole	Letrozole	
19	IDC	Y	Y	Y	Pleural effusion	N/A	N/A	N/A	Docetaxel/ trastuzumab/ pertuzumab, trastuzumab/ pertuzumab/ letrozole	Docetaxel + Trastuzumab + pertuzumab	Docetaxel + Trastuzumab + pertuzumab	

*ALND*: axillary node dissection; *CSF*: cerebrospinal fluid; IBC: inflammatory breast cancer; *IDC*: invasive ductal carcinoma; *ILC*: invasive lobular carcinoma; *IORT*: intraoperative radiation therapy; *PLC*: pleomorphic lobular carcinoma; *LN*: lymph node; *MRM*: modified radical mastectomy; *SLN*: sentinel lymph nodes; *WBRT*: whole brain radiation therapy; *XRT*: radiotherapy; *BMX*: bilateral mastectomy; *N/A*: information not available.

**Fig. 1:**
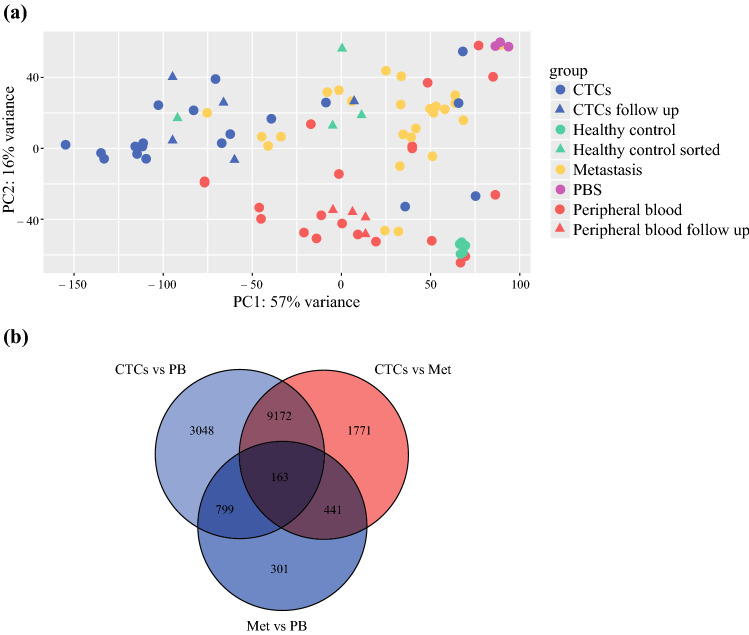
Principal component (PC) analysis and differential gene expression distinguishes different sample types (CTCs, CTCs follow-up, metastases, PB, PB follow-up, healthy controls, healthy controls sorted, PBS). **A** Principal component analysis demonstrating separation of the majority of CTCs versus metastases and PB in PC1 (57% of variance explained), and separation of CTCs and metastases from PB in PC2 (16% of variance explained). **B** Venn diagram for inter-group comparison of differentially expressed genes (FDR adjusted *p* < 0.05)

Principal component analysis (PCA) showed separation of the majority of CTCs versus metastases and PB in PC1, and separation of CTCs and metastases from PB in PC2 (Fig. 1A). A Venn diagram is shown in Fig. 1B for intergroup comparison of gene expression in CTCs, metastases, and PB. A pairwise grouped comparison was used to identify the five most up- and downregulated genes with an expression change of at least 2-fold and adjusted *p*-value of < 0.05: CTCs vs PB downregulated: YTHDC1, CREG1 CLK2, ADIPOR1, RN7SL2; upregulated: GPRC5D, LINC01376, LOC727993, TAS1R3, ARC; CTCs + metastases vs PB downregulated: SNAP23, GALNS, SELENOS, RN7SL2, MORN1; upregulated MGP, GDF9, MYH11, AZGP1, LUM; CTCs vs metastases downregulated: ABCF1, TJP1, DLG5, PTPRK, H19; upregulated: GRPC5D, TMEM198, LOC727993, ARC, RNU6ATAC (Supplementary Fig. S2). In summary, these results show that RNA-Seq can detect distinct gene expression features in enriched CTCs compared with metastases and PB.

### Gene Expression of Potentially Clinically Actionable Genes Relevant to Breast Cancer

**Fig. 2:**
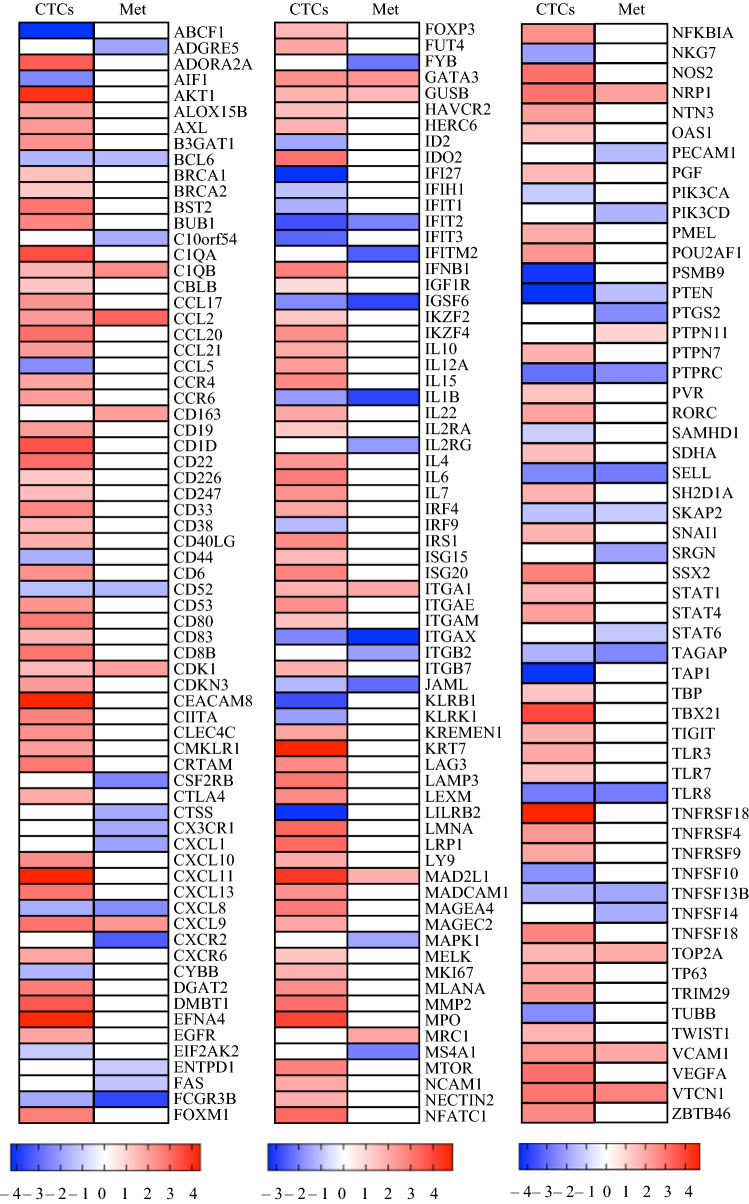
Absolute normalized expression (RPKM) of immune response assay genes in CTCs, metastases and PB. Heatmap showing the expression of immune target genes (*n* = 207) (log fold change) from the Oncomine Immune Response Research Assay in CTCs and metastases normalized to PB based on RNA-Seq data

CTCs showed overall many more differentially expressed immune oncology target genes (Oncomine Immune Response Assay) compared with peripheral blood than did metastatic biopsies (overexpression: CTCs 131 vs metastases 15, 8.7-fold difference; downregulation: CTCs 38 vs metastasis 37, 1.03-fold difference). A total of 12 overexpressed and 15 downregulated genes compared with PB were in common between CTCs and metastasis (Fig. 2A, Supplementary Table S4). Notably, PD-L1 expression was significantly lower in both CTCs and metastases compared with PB (CTCs versus PB *p* = 3.5 × 10^−5^, CTCs vs metastases *p* = 0.004 and metastases versus PB *p* = 0.004) (Supplementary Fig. S3).

**Fig. 3:**
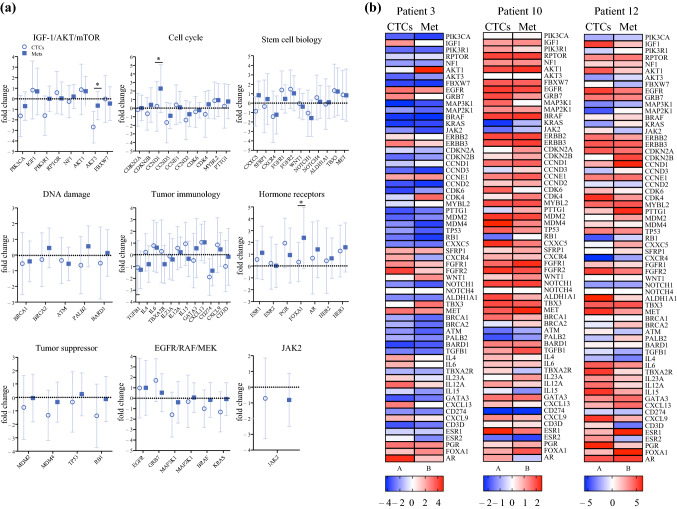
Comparison of potentially clinically actionable breast cancer related genes in CTCs and metastases normalized to PB.** A** Comparison of gene expression between paired CTCs and metastases in 64 clinically actionable genes grouped into 9 biological pathways for 19 patients (*p ≤ 0.05) (2-way ANOVA). **B** Expression of 64 clinically actionable target genes in three representative patient samples (patient numbers correspond to Table 1): expression is shown as log fold change values for each gene in CTCs and matched metastatic biopsies, each normalized to the patient’s own PB

We found concordant expression of 50/64 (78%) potentially clinically actionable target genes in CTCs and corresponding metastases (Fig. 3A). No genes were uniformly overexpressed or downregulated in all sample groups (i.e., CTCs or metastases). Only 3/64 (4.7%) genes showed statistically significantly discordant expression in CTCs vs metastases (AKT3 *p* = 0.018, CCND1 *p* = 0.025, FOXA1 *p* = 0.034) (Fig. 3A). Figure 3B shows representative patient samples (n = 3) for the expression of all 64 clinically actionable target genes with related clinical trials as well as targeted therapeutics. The majority of CTC and metastasis samples showed overexpression of these targetable genes compared with PB, with few exceptions (i.e., lower or weak expression, < 2-fold) (Fig. 3B) (Supplementary Fig. S5, Supplementary Table S1). These results indicated that CTCs could potentially serve as a surrogate for distant macrometastases for the identification of druggable targets.

### Sequential Analysis of CTC Samples

**Fig. 4:**
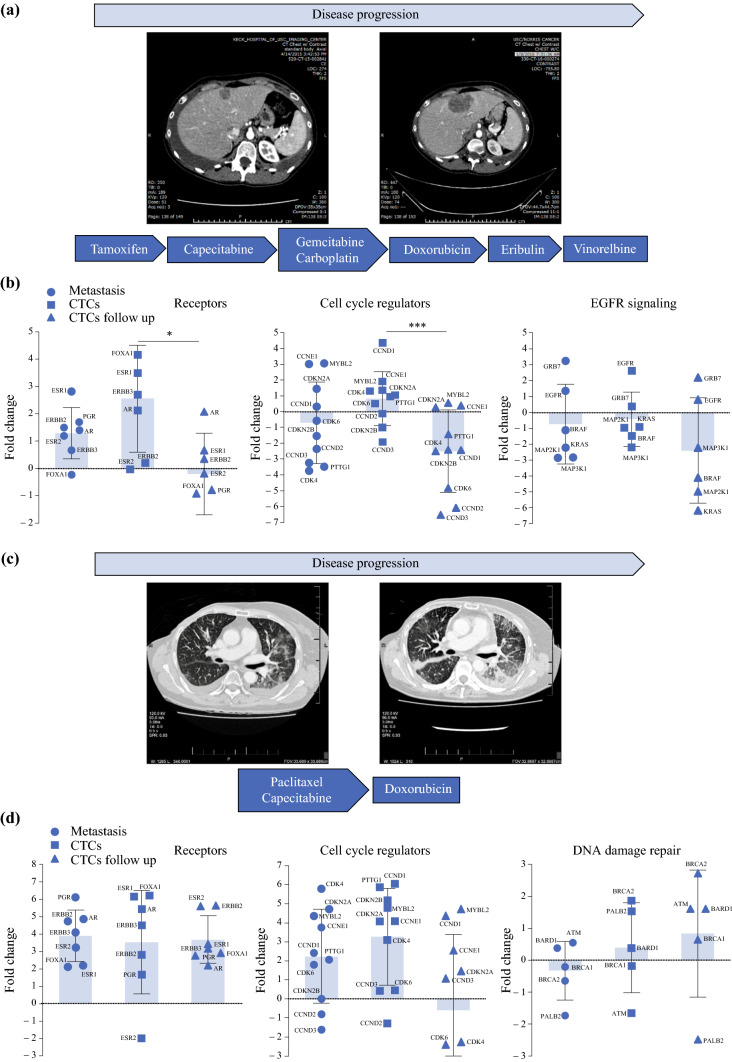
Intra-patient analysis predictive biomarker gene expression in metastatic biopsy tissue and two serial CTC harvest time points. **A**, **C** Clinical data (including treatment and CT imaging studies). **B**, **D** Representative clinically actionable genes and pathways (3 per patient) for CTCs: CTC follow-up samples for 2 patients are shown. The *gray bar* indicates the mean expression and standard deviation for all genes represented in the biological function. Expression of individual genes are represented by the *black squares*. A two-way ANOVA was performed to compare overall expression between all three sample types (metastatic sample, CTCs, CTCs follow-up) (*p = 0.014, ***p = 0.0005)

We tracked four patients with repeated harvest of CTCs at a second time point, as well as obtaining the imaging studies and systemic therapies these patients received (average time between first and second CTC harvest time point was 4 ± 0.8 months). Representative results for two patients are shown in Fig. 4 (the remaining data can be found in Supplementary Fig. S6). The metastatic sites profiled for these patients were pleural effusions (ER/PR+, HER2-) (patient 1 in Table 1) (Fig. 4A and B), (ER+, PR/HER2-) (patient 15 in Table 1) (Fig. 4C and D). For the first patient, hormone receptor genes were downregulated upon sequential CTC assessment after receiving tamoxifen (Fig. 4B) (fold change expression compared with PB: receptors—metastasis 1.29 ± 0.94, CTCs 2.56 ± 1.96, CTCs follow-up −0.2 ± 1.48; cell cycle—metastasis −0.71 ± 2.59, CTCs 0.83 ± 1.7, CTCs follow-up −2.50 ± 2.59; EGFR signaling—metastasis −0.73 ± 2.49, CTCs −0.43 ± 1.71, CTCs follow-up −2.39 ± 3.31). The difference in expression was statistically significant for comparison of CTCs and CTCs follow-up for receptor expression (*p* = 0.014) and cell cycle gene expression (*p* = 0.0005). The follow-up CTC sample in the second patient showed upregulation of DNA-damage repair genes under treatment with an alkylating agent (doxorubicin) (Fig. 4D) (fold change expression compared to PB: receptors—metastasis 3.90 ± 1.48, CTCs 3.54 ± 2.98, CTCs follow-up 3.68 ± 1.38; cell cycle—metastasis 2.24 ± 2.47, CTCs 3.27 ± 2.55, CTCs follow-up −0.59 ± 3.97; DNA damage repair—metastasis −0.33 ± 0.92, CTCs 0.39 ± 1.42, CTCs follow-up 0.84 ± 1.99). For both patients, the CTC follow-up samples showed a reduction in cell cycle gene expression. These data revealed an evolution of biological features within each patient’s disease under therapeutic pressure, with implications for clinical management.

### Somatic Single Nucleotide Variant (SNV) Analysis

**Fig. 5:**
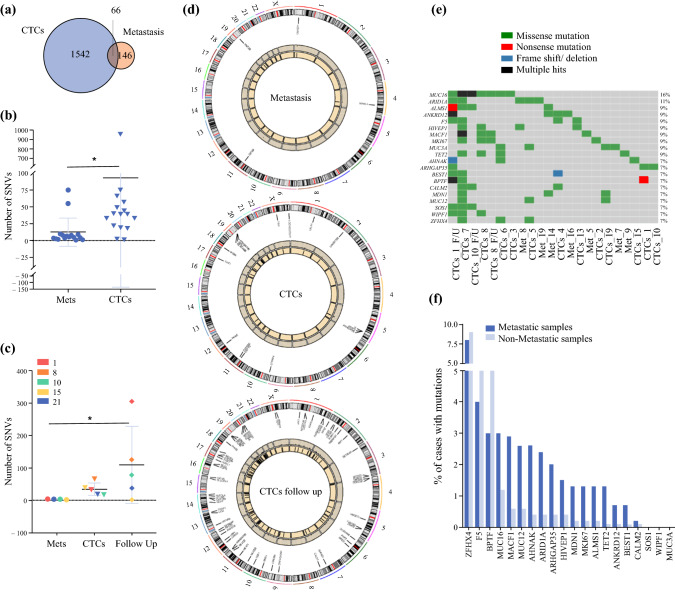
SNVs detected in CTCs and metastases compared with PB. **A** Overlap of all genes with SNVs found across all CTCs and metastases samples. **B** Number of SNVs in individual metastatic samples and CTCs side-by-side (*p = 0.011) (Wilcoxon test). **C** Number of SNVs in 5 samples with CTC follow-up data (patient numbers correspond to Table 1). Each *column* represents a sample type, each *color* represents a patient with ID number (*p = 0.02) (Friedman test) (the SNV for metastasis sample in patient 8 failed). **D** Circos plots showing mutations and corresponding gene expression for one patient (corresponding to patient 10 in Table 1) including CTCs, CTCs follow-up and metastasis. *Innermost circle*: position of the mutated gene on the corresponding chromosome on the *outermost circle. Middle circle*: RPKM value for expression of genes with SNVs. **E** Oncoplot showing driver genes (*n* = 20) with the highest number of mutations across the highest number of samples (percentage of samples with mutations on *right* side of the graph). **F** Validation of mutated driver genes identified by RNA-Seq in publicly available data sets using cBioPortal comparing metastatic (*n* = 396) vs non-metastatic (*n* = 5158) breast cancer data sets (mean number of mutations in metastatic vs non-metastatic samples was 1.94 ± 1.84 vs 1.20 ± 2.35; *p* = 0.009) (Wilcoxon test)

Across all samples, we detected SNVs in 1754 genes (1608 in CTCs, 212 in metastases). At the gene level, 65/212 (31%) of the gene mutations in the metastatic biopsies were also present in the CTCs (Fig. 5A, Supplementary Table S5). A total of 2258 somatic mutations were found across all samples, with CTCs showing a 9.4-fold higher number of SNVs compared with metastases (2041 in CTCs, 217 in metastases; mean and SD: CTCs 93 ± 231 vs metastases 13 ± 21, *p* = 0.01) (Fig. 5B). We detected 344 variants (17%) found in all CTCs and 42 variants (1.9%) in all metastases that corresponded to SNVs found in the COSMIC (Catalogue of Somatic Mutations in Cancer) database (Supplementary Table S6). Our data showed increasing genomic complexity represented by a higher number of SNVs in 3/4 patients with follow-up at the second time point (mean SNVs: metastasis 4 ± 1, CTCs 35 ± 20, CTCs follow-up 110 ± 119, CTCs vs CTCs follow-up *p* = 0.035) (Fig. 5C and D, Supplementary Fig. S7A–C). Sanger sequencing validated 6 of 10 selected RNA-Seq variants (60%) (Supplementary Fig. S7). We ranked the top 20 most frequently mutated genes across our samples: AHNAK, ALMS1, ANKRD12, ARID1A, ARHGAP35, BEST1, BPTF, CALM2, F5, HIVEP1, MACF1, MDN1, MIK67, MUC3A, MUC12, MUC16, SOS1, TET2, WIPF1, ZFHX4 (Fig. 5E, Supplementary Fig. S9, Supplementary Table S8) and validated these genes in publicly available data sets using cBioPortal, comparing metastatic (n = 396) vs non-metastatic (n = 5158) BC samples. Seventeen out of those 20 genes were mutated in BC with VAF varying from 0.2 to 9% (Fig. 5F). We found a significant difference in SNVs between metastatic and non-metastatic cases (*p* = 0.009) (mean metastatic vs non-metastatic 1.94 ± 1.84 vs 1.2 ± 2.35), with up to 10-fold differences for single genes (i.e., TET2). Comparing 184 putative BC driver genes identified in the IntOGen-mutations platform^22^ we found SNVs in 44/184 (24%) in our RNA-Seq data set (Supplementary Table S9). Of these, 34/44 (77.3%) were present only in CTC samples, 4/44 (9.1%) only in metastatic samples and 5/44 (11.4%) in both CTCs and metastases.

In summary, our analysis demonstrated a higher number of heterogeneous somatic mutations in CTCs compared with macrometastatic biopsies, an increase in the number of SNVs in CTCs over time, and revealed that RNA-Seq of CTCs can detect driver gene mutations in MBC.

## Discussion

We present the gene expression profiling of enriched CTCs from MBC patients with comparison to metastases and PB, all acquired prior to treatment or at disease progression prior to a new line of therapy. PCA analysis showed that all sample groups (CTCs, metastases, and PB) separated in PC1, with most CTCs and metastases clustering together in PC2. The partial overlap with PB might be explained by findings that CTCs frequently associate with WBCs, in particular neutrophils.^25^ Our lab has also previously shown that even ultra-pure CTC populations express WBC genes.^21^ Both metastatic and CTC samples (expected 4–10 background leukocytes per CTC after Parsortix enrichment) likely also contain WBCs.

Several genes with biological and clinical implications for BC were highly expressed in CTCs. GPRC5D has been previously associated with tamoxifen resistance.^26^ TMEM198 promotes LRP6 phosphorylation in activating Wnt signaling,^27^ which has been associated with CSC biology in BC. The apoptosis inhibitor ARC has been associated with chemotherapy resistance, tumorigenesis, and metastasis in the polyoma middle T-antigen (PyMT) transgenic mouse model of BC.^28^ It has also been shown to lead to TP53 inactivation in TP53 WT malignancies.^29^ LOC727993, a non-coding antisense RNA of the gene known as PDYN-AS1, and RNU6ATAC, a small nuclear RNA associated with U12-dependent splicing, have not previously been demonstrated to be involved with tumor biology. Our approach identified both known and novel genes associated with CTCs, suggesting that CTCs might be suitable as a discovery tool to better understand the fundamental tumor biology of metastasis.

We found highly concordant expression in potentially clinically actionable genes in corresponding CTC and metastatic samples, demonstrating the potential clinical relevance of CTCs as predictive biomarkers in BC. Nevertheless, we also observed discordant results, which could be due to various conditions: (1) a heterogeneous origin of CTCs from various metastatic sites or seeding of CTCs from the primary tumor site, (2) changes in transcriptional programs once cells “settle” in a new environment, influenced by tissue or site-specific micro-environmental cues,^30^ or (3) differences in the timing of when the metastatic (more remote and established) vs seeding of CTCs occurs (which may be more reflective of recent genomic alterations and treatments). 

Longitudinal analysis of four patients with serial CTC assessments showed changes in biological pathway activation during treatment and disease progression. We found markedly increased genetic complexity in 3 out of 4 patients over time. These results indicate that serial CTC harvest might capture changes in additional mutation burden as a cancer evolves, particularly under the selection pressure of anti-cancer therapies.^31^ Periodic surveying of the mutational evolution using CTCs could thus impact clinical decision making. Additionally, CTCs might capture the mutational landscape of a patient’s cancer from different metastatic sites more comprehensively than single site biopsies.^32^

Compared with gene expression of potentially actionable genes, we observed a much lower concordance in our SNV analysis. As there is no standard tool or pipeline for using RNA-Seq to call SNVs, we established a workflow for the purpose. SNV calling decreases with low read depth or low allelic frequency, diminishing the sensitivity of SNV detection.^33^ The lack of overlap and greater genomic complexity in CTCs could also represent the pool of heterogeneous somatic mutations from various metastatic sites and different cancer cell clones compared with individual metastatic sites. The strength of our approach is the inference of expressed mutations, given that not all DNA mutations are expressed. The finding that lncRNAs were frequently mutated in CTCs and metastases offers an interesting opportunity to further investigate the role of regulatory RNAs in metastasis.^34^ As this aspect of our paper is the most speculative piece of the manuscript, we believe that better tools are needed for more sensitive SNV calling from complex samples such as circulating tumor cells. Single cell sequencing studies may shed light on this by controlling for the input of cancer vs peripheral blood mononuclear cells.

Analysis of the expression of IO target genes demonstrated that CTCs expressed 170/200 genes related to immune response while metastatic biopsies expressed only 52/200 such genes, suggesting an important role for the immune system in CTC biology and potential immune escape. During dissemination, CTCs are exposed to many types of stress in the blood microenvironments and direct exposure to immune surveillance. Our results are in line with previous studies demonstrating upregulation of potential immune-escape mechanisms.^35^ Several highly expressed IO genes in CTCs in our study have been shown to play important roles in immune evasion and metastatic efficiency: AKT1 can potentially suppress immunodetection by activating myeloid suppressor cells.^36^ The complement component C1q might facilitate the metastatic potential of CTCs.^37^ CXCL9-11 might act as a double-edged sword via paracrine and autocrine signaling or interaction with PD-L1, inhibiting or facilitating immune escape and metastatic seeding, respectively.^38^ We found expression of PD-L1 to be lower in CTCs and metastases compared with PB samples in our study. Although this marker has been suggested as a potential biomarker in CTCs,^39^ our study differs regarding the detection method and CTC capture platform with consideration of gene expression relative to background PB. High gene expression of PD-L1 in PBMCs is to be expected; the *Human Protein Atlas* shows high expression of CD274 (PD-L1) in basophils.^40^ These findings have implications for the use of immune targeted drugs and warrant further investigation into immune targeting of CTCs.^41^

There are several limitations of our study such as a relatively small number of patients, and CTC enrichment purity. We applied per patient normalization, utilizing matched white blood cells as a pre-specified analysis plan regarding our primary research question of comparing the gene expression of CTCs vs metastases for a list of well characterized, potentially clinically actionable marker genes. Our strategy of per patient normalization to PB signal is novel, focusing attention on genes with strong differential expression between tumor and blood by controlling for leukocyte background.^21,42^ Thus, subtle differences in gene expression might not be captured with our method. Ideally, sequencing of pure cell populations, even at single cell level should be attempted to characterize differences of gene expression between CTCs and WBCs more stringently.^43^

For gene expression results, standard normalization (reported as reads per kilobase of transcript, per million mapped (RPKM)) was applied but, due to the nature of our approach, we cannot rule out a certain degree of amplification bias. However, we previously utilized unspiked negative controls and extensively validated our RNA amplification strategy.^21,44^ Although we successfully detected SNVs, our current data analysis pipeline does not allow for the detection of copy number variation. SNV-calling from RNA-Seq is less well established compared with the DNA based method, and further validation will be needed in the future.

## Conclusions

RNA-Seq of Parsortix-enriched CTCs could lead to minimally invasive, real-time diagnostic strategies for precision therapeutic decision making for MBC patients. Our approach could serve as a surrogate liquid biopsy for potentially clinically actionable drug target gene expression and mutations, allowing longitudinal assessment of the evolution of a patient’s cancer.

## Supplementary Information

Below is the link to the electronic supplementary material.

Supplementary file1 (DOCX 31951 kb)

Supplementary file2 (DOCX 94 kb)

Supplementary file3 (XLSX 161 kb)

Supplementary file4 (XLSX 210 kb)

Supplementary file5 (DOCX 26 kb)
