# Correction to: Circulating Tumor Cell Transcriptomics as Biopsy Surrogates in Metastatic Breast Cancer

**DOI:** 10.1245/s10434-022-11589-y

**Published:** 2022-03-08

**Authors:** Alexander Ring, Daniel Campo, Tania B. Porras, Pushpinder Kaur, Victoria A. Forte, Debu Tripathy, Janice Lu, Irene Kang, Michael F. Press, Young Ju Jeong, Anson Snow, Yue Zhu, Gabriel Zada, Naveed Wagle, Julie E. Lang

**Affiliations:** 1 Division of Surgical Oncology, Department of Surgery and University of Southern California Norris Cancer Center, University of Southern California, Los Angeles, CA USA; 2 Present Address: Department of Hematology and Medical Oncology, University Hospital Zurich, Zurich, Switzerland; 3 Department of Biological Sciences, University of Southern California, Los Angeles, CA USA; 4 Division of Medical Oncology, Department of Medicine, SUNY Downstate Medical Center, New York, NY USA; 5 Department of Breast Medical Oncology, UT MD Anderson Cancer Center, Houston, TX USA; 6 Division of Medical Oncology, Department of Medicine and University of Southern California Norris Cancer Center, University of Southern California, Los Angeles, CA USA; 7 Department of Pathology and University of Southern California Norris Cancer Center, University of Southern California, Los Angeles, CA USA; 8 Department of Surgery, Catholic University of Daegu School of Medicine, Daegu, Republic of Korea; 9 Department of Neurosurgery and University of Southern California Norris Cancer Center, University of Southern California, Los Angeles, CA USA; 10 Present Address: Division of Breast Services, Department of General Surgery, Cleveland Clinic Breast Cancer Program, Cleveland, OH USA

## Correction to: Ann Surg Oncol 10.1245/s10434-021-11135-2

The original online version of this article was revised. The last paragraph of the abstract has been deleted.

