# Supplementary material for: Circulating Tumor Cell Transcriptomics as Biopsy Surrogates in Metastatic Breast Cancer
Source: Ann Surg Oncol. 2022 Jan 9;29(5):2882–94. doi: 10.1245/s10434-021-11135-2 (PMC8989945; doi:10.1245/s10434-021-11135-2)
Supplement: Supplementary file 1 — Supplementary file1 (DOCX 31951 kb) [file 10434_2021_11135_MOESM1_ESM.docx]

Supplementary figures

**Supplementary figure S1.** **Isolation and quantification of spike in BC cells (MDA-MB-231) using the ANGLE Parsortix device. 1A:** 3.5 ml of peripheral blood form healthy donors were spiked with 100 GFP labeled BC cells. **1B:** The samples were sorted using microfluidic cassettes with different critical step sizes (4.4, 6.5, 8.0 or 10.0 µm). **1C:** GFP positive cells were subsequently counted using an inverted fluorescent microscope

**Supplementary figure S2A-D.** **Volcano plots showing differential gene expression based on grouped comparison:** **1A:** CTCs vs. PB. **S1B:** CTCs vs. Metastases **S1C:** CTCs plus Metastases vs. PB. **S1D:** Metastases vs. PB.

**Supplementary figure S3:** **PD-L1 expression: Cumulative RNA expression of PD-L1 in CTCs, metastatic samples and PB:** mean expression CTCs: 0.5117863 ± 0.5917061, mean Met: 1.377844 ± 1.613414, PB: 5.929317 ± 8.053711. CTCs vs. metastases *p*-value = 0.004254, CTCs vs. PB *p*-value = 3.517e-05, metastases vs. PB *p*-value = 0.004002.

**Supplementary figure S3. Scatter plot of CD45 and EpCAM distribution:** All Samples in our analysis were plotted according to the mRNA-expression of the WBC marker CD45 on x-axis and the epithelial cell marker EpCAM and the y-axis.

**Supplementary figure S4. Potentially clinically actionable breast cancer related genes that were differentially expressed CTCs and metastases compared to PB.** Expression of n=64 potentially clinically actionable genes between matched CTCs and metastases (data for n=16 patients shown here).

**Supplementary figure S6-1 to4A and B: Intra-patient (n=3) two time-point comparison: A:** Clinical data (including treatment and CT imaging studies) and sample collection (top panels). **B:** Differences in the expression of 64 clinically actionable genes bundled into 9 functional categories between metastasis and two CTC harvest times points. indicates transcription regulator, upright ellipse indicates transmembrane receptor, diamond indicates enzyme, triangle indicates kinase, trapezoid indicates transporter, solid circle indicates ‘other’).

**Supplementary figure S7A-C.** **Circos plots for three additional patients with follow up CTCs.** Circos plots showing mutations and corresponding gene expression for one patient including CTCs, CTCs follow up and metastasis. Innermost circle: Position of the mutated gene on the corresponding chromosome on the outermost circle. Middle circle: RPKM value for expression of 100 genes with SNVs.

**Supplementary figure. S8. Sanger sequencing of SNVs found in RNA-Seq of CTCs and metastases: A:** Mutations confirmed via Sanger sequencing: *TRIM25 (CTCs)* TCG>T chr17: 56,901,561, *MUC5B (CTCs)* CCG>C chr11: 1,245,888 and *ZMIZ1 (CTCs)* C>A chr10: 79,298,435. **B**: discrepancy between Sanger sequencing and RNA-Seq: *JAK2* (met) chr9: 5,066,709, *STAT4* (CTCs) chr2: 191,073,177, *DHRS9* (met) chr2: 169,083,565, *CDK12* (CTCs) chr17: 39,471,212. ^ changes found in RNA-Seq data.

**Supplementary** **figure S9. Driver gene mutation analysis breakdown. A:** Variant classification. **B:** Variant type. **C:** SNV Class (T – thymine, A – adenine, G – guanine, C – cytosine). **D:** Number of variants (y-axis) per sample (x-axis). **E:** Variant classification summary (color coding same as in 7A). **F:** Top 10 mutated genes.
