# Supplementary material for: Circulating Tumor Cell Transcriptomics as Biopsy Surrogates in Metastatic Breast Cancer
Source: Ann Surg Oncol. 2022 Jan 9;29(5):2882–94. doi: 10.1245/s10434-021-11135-2 (PMC8989945; doi:10.1245/s10434-021-11135-2)
Supplement: Supplementary file 2 — Supplementary file2 (DOCX 94 kb) [file 10434_2021_11135_MOESM2_ESM.docx]

Supplementary tables

**Supplementary table S1:** List of PCR primer sequences and Sequencing Primers used for Sanger sequencing

| **Gene** | **Forward PCR Primer** | **Reverse PCR Primer** | **Sequencing primer** |
| --- | --- | --- | --- |
| ROR1 | TCAGCCTCTCTGTGATCCCA | GTGTCTGTGGTGTCGAGGTT | TCAGCCTCTCTGTGATCCCA |
| ZMIZ1 | TCTTCTGAGCAGGACAGAGA | GGAAGGGTGGTTTGACGTCT | TGCGGTGTATGTCCATAGCC |
| JAK1 | CAGTCAGCGTGTTTTGGTCG | CAGATCGAGGTGCAGAAGGG | CAGTCAGCGTGTTTTGGTCG |
| TRIM25 | TGCTCTGGTGGATGCCTTTT | GGACTTAGCACTCTTGGGCA | TGCTCTGGTGGATGCCTTTT |
| STAT4 | TCACATGGATAGAGCATCAGACA | AGAGCAAGGAAGGCTGTAGT | TCACATGGATAGAGCATCAGACA |
| DHRS9 | TGGACTCATCAGTGTGTGACACT | GCATTCAGTAAACGTTTGCAGC | TGGACTCATCAGTGTGTGACACT |
| CDK12 | GCTGCAGCAAAGATGGATGG | CTCCTCTTTCAGTGCTATGGGT | GCTGCAGCAAAGATGGATGG |
| MUC5B | GCAGGTGGGGAAGTTCAAGA | GGATTTGGTGGCTGTGCTTG | GCAGGTGGGGAAGTTCAAGA |
| JAK2 | TGTCCCTTGAAGTGGTTTGAAC | ACCTCGACAGCAAAAGTCAA | TGTCCCTTGAAGTGGTTTGAAC |

**Supplementary table S2.** List of potentially clinically actionable breast cancer related genes. A total of 64 genes were curated from relevant literature and included if clinical and preclinical therapeutic, prognostic or diagnostic implications were available.

| Genes | Clinical trials and references | Therapeutic agents |
| --- | --- | --- |
| CCND1 ^fm^ | NCT02936206, NCT03304080, NCT01740427, NCT02187783 | Ribociclib, palbociclib, abemaciclib |
| CCND2 ^fm^ | NCT01037790, NCT00334542, NCT02187783, NCT01037790 | Ribociclib, palbociclib, abemaciclib, PD 0332991 |
| CCND3 ^fm^ | NCT02187783 | Ribociclib, palbociclib, abemaciclib |
| CCNE1 ^fm^ | NCT03184090 | Ribociclib, palbociclib, abemaciclib |
| CDK6 ^fm^, CDK4 ^fmo^ | NCT02187783, NCT01037790 | Ribociclib, palbociclib, abemaciclib, PD 0332991 |
| CDKN2A ^fmo^, CDKN2B ^fm^ | NCT01740427 | Ribociclib, palbociclib, abemaciclib |
| MDM2 ^fmo^ | NCT02579824, NCT00559533, NCT01877382 | DS-3032b, RG-7112, Nutlin-3, NSC207895 |
| MDM4 ^fm^ | NCT01877382 | RG-7112, Nutlin-3 |
| MYBL2 | NCT01273415 | Cytotoxic chemotherapy |
| PTTG1 | PMID 29078751, 24395789, 25739119 | Immunotherapy |
| RB1 ^fm^ | NCT02599363, NCT03130439, NCT03007979 | Ribociclib, abemaciclib, palbociclib |
| TP53 ^fm^ | NCT00044993, NCT00004038, PMID 19731257, PMID 10572598, NCT01386502, NCT00496860 | Gamboric Acid, YK-3-237, PRIMA-1, Ad5CMV-p53, p53 genetic vaccine, ALT-801 |
| AR ^fm^ | NCT01889238, NCT01918306, NCT02457910, NCT01151046, NCT03207529, NCT02130700, NCT01990209, NCT02599363, NCT03130439, NCT03007979 | Enzalutamide, bicalutamide, MK-2866, flutamide, dehydroepiandrosterone (DHEA), alpelisib, VT-464, GTx-024, orteronel, ribociclib, abemaciclib, palbociclib |
| ESR1 ^mo^ | NCT00849030, NCT03455270, NCT02650817, NCT02734615, | AZD9496, fulvestrant, tamoxifen, letrozole, anastrozole, exemestane, G1T48, RAD1901, LSZ102, BYL719 |
| ESR2 | NCT00580112, NCT00050427, NCT020898547, NCT02067741 | Trabectedin, toremifene, tamoxifen, CR1447 |
| FOXA1 | PMID 28270510, 27185372, 21151129 | Cancer stem cell targeting agents, decreased response to fulvestrant, tamoxifen, letrozole, anastrozole, exemestane |
| PGR | NCT00849030, NCT01151046, NCT01421472, NCT03241810 | AZD9496, fulvestrant, tamoxifen, letrozole, anastrozole, exemestane, seribantumab (MM-121) |
| GATA3 ^fm^ | NCT00897065, PMID 28581515 | Tamoxifen, letrozole |
| CD3D | PMID 22151962 | JNJ-7706621 |
| CXCL13 | PMID 29077781 | Neo-adjuvant chemotherapy |
| CXCL9 | PMID 19917869, NCT03112590 | INF gamma |
| IL12A  IL15 | NCT00004074  NCT03175666, NCT03127098 | ABI-007, ALT-803 |
| IL23A | PMID 28470472 | Guselkumab |
| IL4 | NCT00039052 | Interleukin-4 PE38KDEL cytotoxin (NBI-3001) |
| IL6 | NCT03135171, NCT02041429 | Tocilizumab, ruxolitinib |
| TBXA2R | PMID 27487152, NCT02538471, NCT00821964 | LY2157299, imiquimod and abraxane |
| TGFB1 | NC01401062 | Fresolimumab |
| ATM ^fmo^, BARD1 ^fm^ | NCT02401347, NCT03344965 | Talazoparib, olaparib |
| BRCA1 ^fmo^, BRCA2 ^fmo^ | NCT02163694, NCT01506609, NCT02032823, NCT03205761, NCT02681562, NCT03150576, NCT02826512, NCT01905592 | Olaparib, niraparib, talazoparib, velaparib |
| PALB2 ^fm^ | NCT02401347, NCT03344965 | Olaparib, talazoparib |
| AKT1 ^fmo^, AKT3 ^fm^ | NCT01964924, NCT02162719, NCT01226316, NCT02077569, NCT01277757, NCT02423603, NCT01980277, NCT01964924, NCT01992952 | Ipatasertib, AZD5363, PF-04691502, triciribine, CCT128930, Honokiol, AT13148, TIC10 (ONC201), MK2206, LY2780301, GSK2141h95 |
| BRAF ^mo^ | NCT02401347, NCT03065387, NCT01363232, NCT01337765 | Cobimetinib, vemurafenib, dabrafenib, trametinib, BKM120 Plus MEK162, BEZ235 Plus MEK162 |
| EGFR ^fmo^ | NCT02465060, NCT01582191, NCT01934335, NCT01732276, NCT00739063, NCT02720185, NCT00820924, NCT0089450 | Afatinib, erlotinib, gefitinib, osimertinib, vandetanib, dasatinib, lapatinib, panitumumab |
| ERBB2 ^fmo^ | NCT01772472, NCT03065387, NCT00878709, NCT01953926, NCT00875979 | Trastuzumab emtansine, lapatinib, trastuzumab, pertuzumab, neratinib |
| ERBB3 ^m^ | NCT03065387, NCT00073528, NCT02980341, NCT02297698, NCT01918254, NCT03321981, NCT00073528 | Neratinib, GW572016, U3-1402, HER2 vaccine nelipepimut-S, lumretuzumab, MCLA-128, NCT02912949 |
| FBXW7 ^fm^ | PMID 23834147, PMID 24899509, PMID 22389469 | SINE KPT-185, Oridonin, Genistein |
| GRB7 | PMID 17426702 | G7-18NATE-P |
| IGF1 ^fm^ | NCT00984490, NCT02278965, NCT01479179, NCT00984490, NCT00759785, NCT01372618, NCT00897884 | tivozanib, AMG 479, metformin, MK-0646, pasireotide, ganitumab |
| JAK2 ^fmo^ | NCT02041429, PMID 20111893, NCT02637375, NCT01929941 | Ruxolitinib, ganetespib, INCB047986 |
| KRAS ^fmo^ | NCT00463788, NCT00894504, NCT00463788, NCT02259114, NCT01520389, NCT01337765 | Cetuximab, panitumumab, regorafenib, OTX105/MK-8628, MM-151, BEZ235 plus MED162 |
| MAP2K1 ^fmo^  MAP3K1 ^fm^ | NCT02322814, NCT01160718, NCT02685657, NCT00147550, NCT01467310, NCT01337765, NCT01964924, PMID24386504 | Cobimetinib, trametinib, AZD6244, MSC1936369B, SELUMETINIB, PD-325901, GSK1120212, MEK162 |
| NF1 ^fmo^ | NCT02583542 | Selumetinib |
| PIK3CA ^fmo^, PIK3CB ^fm^,  PIK3R1 ^m^ | NCT01923168, NCT02437318, NCT00310180, NCT02705859, NCT03006172, NCT02340221, NCT01918306, NCT02465060, NCT03337724, NCT01513356, NCT01337765, NCT01928459, NCT03243331 | Buparlisib, alpelisib, serabelisib, copanlisib, GDC-0077, taselisib, GDC-0941, ipatasertib, BKM120, BEZ235, BGJ398 with BYL719, gedatolisib |
| RPTOR | NCT02456857, NCT01674140,  NCT00107016, NCT02465060, NCT02583542, NCT01390818, NCT01337765 | Everolimus, AZD8055, bevacizumab, voxtalisib, PP242, OSI-027, apitolisib, gedatolisib (PKI-587), sapanisertib, AD6244, SAR245409, BEZ235 |
| ALDH1A1 | NCT01190345, NCT01424865, NCT00949013, NCT01688609, NCT02001974, NCT01372579 | Trastuzumab, bevacizumab, doxorubicin and cyclophosphamide, lapatinib, reparixin, eribulin mesylate and carboplatin |
| CXCR4 | PMID 27669438, NCT02327442 | LY2510924, 68Ga-NOTA-NFB |
| CXXC5 | PMID 29627878 | BMD4722 |
| FGFR1 ^fm^, FGFR2 ^fm^ | NCT01283945, NCT02053636, NCT01791985, NCT02052778, NCT02393248 | Lucitanib, AZD4547, TAS-120, INCB054828, NCT01928459, BGJ398 with BYL719 |
| MET ^fmo^ | NCT02465060, NCT03316586, NCT01837602, NCT01575522, NCT01138384 | Crizotinib, cabozantinib, capmatinib, cMet CAR T Cells, tivantinib, foretinic |
| NOTCH1 ^fm^  NOTCH4 ^m^ | NCT02299635, NCT01208441, NCT00645333, NCT01372579, NCT00106145, NCT01151449, NCT01071564, NCT00645333, NCT01372579 | PF-03084014, eribulin mesylate and carboplatin, MK0752, RO4929097 |
| SFRP1 | PMID 28984209 |  |
| TBX3 | PMID 21325450 |  |
| WNT1 | NCT03243331, PMID 20530697, NCT01351103 | LGK974, gedatolisib |

f: included in the Foundation One gene list 2017; m: included in the MSK-IMPACT gene list; o: included in OncoKB level.

**Supplementary table S3.** Sequencing coverage data for all samples

| Sample | Uniquely mapped reads (%) | Total reads | Read length (bp) | Coverage |
| --- | --- | --- | --- | --- |
| 101738-CTCs_S18 | 77.16 | 32,677,715 | 100 | 218 |
| 101738-PB_S12 | 89.71 | 25,309,271 | 100 | 168 |
| 101738-met_S15 | 88.48 | 42,483,481 | 100 | 283 |
| 101738_CTC_Follow-up_S2 | 77.32 | 60,817,403 | 100 | 405 |
| 101738_PB_Follow-up_S3 | 84.86 | 70,851,407 | 100 | 472 |
| 111951-ANGLE-sorted_S2 | 58.18 | 194,957,38 | 100 | 130 |
| 111951-PB_S7 | 76.46 | 31,808,368 | 100 | 212 |
| 111956-ANGLE-sorted_S3 | 3.89 | 1,261,597 | 100 | 8 |
| 111956-PB_S8 | 80.75 | 37,290,944 | 100 | 248 |
| 111958-ANGLE-sorted_S4 | 72.25 | 19,704,388 | 100 | 131 |
| 111958-PB_S9 | 81.33 | 32,640,992 | 100 | 217 |
| 111961-ANGLE-sorted_S5 | 86.24 | 32,960,724 | 100 | 220 |
| 111961-PB_S10 | 77.86 | 29,622,060 | 100 | 197 |
| 112165_CTCs_S7 | 64.91 | 22,165,019 | 100 | 147 |
| 112165_PB_S2 | 82.22 | 35,875,447 | 100 | 239 |
| 112165_met_LN_S4 | 89.77 | 39,593,201 | 100 | 263 |
| 112165_met_lung_S5 | 88.89 | 40,926,265 | 100 | 272 |
| 112370_CTCs_S3 | 67.19 | 21,033,330 | 100 | 140 |
| 112370_MET2_S4 | 88.08 | 82,313,215 | 138 | 757 |
| 112370_MET_S2 | 86.94 | 35,423,022 | 100 | 236 |
| 112370_PB_S1 | 82.91 | 35,239,979 | 100 | 234 |
| 113059_CTC_S11 | 77.32 | 60,622,645 | 100 | 404 |
| 113059_MET_S9 | 82.94 | 50,321,188 | 100 | 335 |
| 113059_PB_S12 | 87.27 | 69,879,922 | 100 | 465 |
| 113166_CTC_S9 | 62.9 | 41,304,654 | 100 | 275 |
| 113166_MET_S12 | 86.29 | 59,598,973 | 100 | 397 |
| 113166_PB_S10 | 83.53 | 65,874,631 | 100 | 439 |
| 113457_CTC_S13 | 78.76 | 68,107,519 | 100 | 454 |
| 113457_MET_S15 | 89.02 | 41,636,737 | 100 | 277 |
| 113457_PB_S14 | 82.93 | 37,201,841 | 100 | 248 |
| 113488_CTC_S6 | 79.35 | 42,829,356 | 100 | 285 |
| 113488_MET_S5 | 87.48 | 38,761,576 | 100 | 258 |
| 113488_PB_S4 | 85.16 | 104,157,542 | 100 | 694 |
| 19065-CTC_Follow-up_S7 | 82.86 | 70,857,329 | 100 | 472 |
| 19065-PB_Follow-up_S8 | 85.26 | 68,141,539 | 100 | 454 |
| 19065_CTC_S16 | 78.47 | 37,815,932 | 100 | 252 |
| 19065_MET1_S10 | 85.74 | 55,212,920 | 100 | 368 |
| 19065_MET2_S11 | 87.47 | 44,444,636 | 100 | 296 |
| 19065_PB_S1 | 84.18 | 74,345,417 | 100 | 495 |
| 28089-CTCs_S17 | 52.91 | 14,767,858 | 100 | 98 |
| 28089-PB_S11 | 84.82 | 42,818,682 | 100 | 285 |
| 28089-met_S14 | 80.79 | 30,267,206 | 100 | 202 |
| 36541_CTC_Follow-up_S7 | 74.44 | 30,653,540 | 100 | 204 |
| 36541_CTCs_S6 | 46.8 | 14,412,047 | 100 | 96 |
| 36541_PB_Follow-up_S2 | 86.29 | 74,846,623 | 146 | 728 |
| 36541_PB_S1 | 82.86 | 32,716,732 | 100 | 218 |
| 36541_met_breast_S3 | 86.06 | 38,645,578 | 100 | 257 |
| 36978_CTC_Follow-up_S6 | 66.61 | 36,423,774 | 100 | 242 |
| 36978_CTC_merged | 6.98 | 2,399,337 | 100 | 16 |
| 36978_LN_merged | 83.14 | 36,605,483 | 100 | 244 |
| 36978_PB_Follow-up_S1 | 86.53 | 52,884,902 | 144 | 507 |
| 36978_PB_merged | 76.82 | 28,906,448 | 100 | 192 |
| 36978_tumor_merged | 81.66 | 41,947,037 | 100 | 279 |
| 68185_CTC_merged | 33.68 | 12,612,731 | 100 | 84 |
| 68185_Met_merged | 75.57 | 33,478,884 | 100 | 223 |
| 68185_PB_merged | 61.23 | 25,198,952 | 100 | 167 |
| 78534-ANGLE-sorted_S1 | 27.26 | 10,497,733 | 100 | 70 |
| 78534-PB_S6 | 78.19 | 32,215,000 | 100 | 214 |
| 78536_CTC_merged | 23.29 | 7,557,947 | 100 | 50 |
| 78536_Met_merged | 81.17 | 27,166,376 | 100 | 181 |
| 78536_PB_merged | 57.8 | 18,236,161 | 100 | 121 |
| 78908_CTC_merged | 90.81 | 16,930,569 | 100 | 112 |
| 78908_Met_merged | 84.56 | 25,863,624 | 100 | 172 |
| 78908_PB_merged | 90.12 | 13,981,377 | 100 | 93 |
| 79388_CTC_S8 | 77.75 | 41,544,629 | 100 | 276 |
| 79388_MET_S5 | 84.74 | 69,930,045 | 100 | 466 |
| 79388_PB_S3 | 86.49 | 83,272,253 | 142 | 788 |
| 79412-follow-up-CTCs_S10 | 23.79 | 5,389,734 | 100 | 36 |
| 79412_2nd_CTCs_S10 | 76.47 | 26,666,798 | 100 | 177 |
| 79412_2nd_PB_S8 | 83.12 | 32,254,804 | 100 | 215 |
| 79412_2nd_met_PE_S9 | 81.7 | 29,864,834 | 100 | 199 |
| 79412_CTC_merged | 1.92 | 632,331 | 100 | 4 |
| 79412_Met_merged | 88.26 | 29,976,921 | 100 | 200 |
| 79412_PB_merged | 79.55 | 26,967,829 | 100 | 180 |
| 79555_CTC_merged | 62.08 | 20,731,115 | 100 | 138 |
| 79555_Met_merged | 93.11 | 24,650,620 | 100 | 164 |
| 79555_PB_merged | 54.53 | 17,619,368 | 100 | 117 |
| 79556_CTC_merged | 3.61 | 1,214,882 | 100 | 8 |
| 79556_Met_merged | 82.92 | 25,357,055 | 100 | 169 |
| 79556_PB_merged | 80.09 | 43,331,065 | 100 | 289 |
| 79644-CTCs_S19 | 50.04 | 14,870,787 | 100 | 99 |
| 79644-PB_S13 | 79.11 | 29,548,610 | 100 | 196 |
| 79644-met_S16 | 77.38 | 21,927,530 | 100 | 146 |
| 80541_CTC_merged | 30.22 | 11,350,353 | 100 | 75 |
| 80541_Met_merged | 66.45 | 26,686,618 | 100 | 177 |
| 80541_PB_merged | 69.21 | 17,419,175 | 100 | 116 |
| 81103_CTC_merged | 83.08 | 38,381,204 | 100 | 256 |
| 81103_Met_merged | 80.07 | 51,411,123 | 100 | 343 |
| 81103_PB_merged | 84.73 | 48,342,285 | 100 | 322 |
| PBS1_S7 | 0.17 | 62,493 | 100 | 0 |
| PBS2_S8 | 0.44 | 152,235 | 100 | 1 |
| PBS3_S9 | 0.25 | 93,928 | 100 | 0 |

**Supplementary table S4:** Oncomine™ Immune Response Research Assay gene list queried for RNA-Seq analysis (n=194)

| Gene | CTC vs PB | Met vs PB | Genes | CTC vs PB | Met vs PB | Gene | CTC vs PB | Met vs PB | Gene | CTC vs PB | Met vs PB | Gene | CTC vs PB | Met vs PB | Gene | CTC vs PB | Met vs PB |
| --- | --- | --- | --- | --- | --- | --- | --- | --- | --- | --- | --- | --- | --- | --- | --- | --- | --- |
| ABCF1 | -4.28 | 0.00 | CD80 | 2.71 | 0.00 | HAVCR2 | 1.53 | 0.00 | KLRB1 | -2.78 | 0.00 | PMEL | 1.87 | 0.00 | TP63 | 1.94 | 0.00 |
| ADGRE5 | 0.00 | -1.86 | CD83 | 1.58 | 0.00 | HERC6 | 1.75 | 0.00 | KLRK1 | -1.58 | 0.00 | POU2AF1 | 2.38 | 0.00 | TRIM29 | 2.30 | 0.00 |
| ADORA2A | 3.21 | 0.00 | CD8B | 2.78 | 0.00 | ID2 | -1.47 | 0.00 | KREMEN1 | 2.12 | 0.00 | PSMB9 | -4.18 | 0.00 | TUBB | -2.41 | 0.00 |
| AIF1 | -2.46 | 0.00 | CDK1 | 1.48 | 1.97 | IDO2 | 3.11 | 0.00 | KRT7 | 4.85 | 0.00 | PTEN | -4.41 | -1.44 | TWIST1 | 1.68 | 0.00 |
| AKT1 | 4.01 | 0.00 | CDKN3 | 2.06 | 0.00 | IFI27 | -3.39 | 0.00 | LAG3 | 2.66 | 0.00 | PTGS2 | 0.00 | -2.43 | VCAM1 | 2.38 | 1.97 |
| ALOX15B | 1.95 | 0.00 | CEACAM8 | 4.35 | 0.00 | IFIH1 | -1.04 | 0.00 | LAMP3 | 3.06 | 0.00 | PTPN11 | 0.00 | 1.01 | VEGFA | 3.12 | 0.00 |
| AXL | 2.12 | 0.00 | CIITA | 2.57 | 0.00 | IFIT1 | -1.32 | 0.00 | LEXM | 2.61 | 0.00 | PTPN7 | 1.77 | 0.00 | VTCN1 | 3.00 | 2.76 |
| B3GAT1 | 2.23 | 0.00 | CLEC4C | 2.25 | 0.00 | IFIT2 | -2.74 | -2.02 | LILRB2 | -3.25 | 0.00 | PTPRC | -2.95 | -2.44 | ZBTB46 | 2.61 | 0.00 |
| BCL6 | -1.57 | -1.50 | CMKLR1 | 2.00 | 0.00 | IFIT3 | -2.44 | 0.00 | LMNA | 3.31 | 0.00 | PVR | 1.39 | 0.00 |  |  |  |
| BRCA1 | 1.33 | 0.00 | CRTAM | 2.72 | 0.00 | IFITM2 | 0.00 | -2.59 | LRP1 | 3.30 | 0.00 | RORC | 2.07 | 0.00 |  |  |  |
| BRCA2 | 1.14 | 0.00 | CSF2RB | 0.00 | -2.51 | IFNB1 | 2.85 | 0.00 | LY9 | 1.91 | 0.00 | SAMHD1 | -1.11 | 0.00 |  |  |  |
| BST2 | 2.78 | 0.00 | CTLA4 | 1.75 | 0.00 | IGF1R | 0.87 | 0.00 | MAD2L1 | 4.29 | 1.85 | SDHA | 1.48 | 0.00 |  |  |  |
| BUB1 | 2.47 | 0.00 | CTSS | 0.00 | -1.78 | IGSF6 | -1.90 | -2.92 | MADCAM1 | 2.48 | 0.00 | SELL | -2.53 | -2.75 |  |  |  |
| C10orf54 | 0.00 | -1.73 | CX3CR1 | 0.00 | -1.81 | IKZF2 | 1.28 | 0.00 | MAGEA4 | 2.96 | 0.00 | SH2D1A | 1.76 | 0.00 |  |  |  |
| C1QA | 3.48 | 0.00 | CXCL1 | 0.00 | -1.93 | IKZF4 | 2.54 | 0.00 | MAGEC2 | 2.05 | 0.00 | SKAP2 | -1.35 | -1.17 |  |  |  |
| C1QB | 1.58 | 2.33 | CXCL10 | 2.36 | 0.00 | IL10 | 1.94 | 0.00 | MAPK1 | 0.00 | -1.46 | SNAI1 | 1.76 | 0.00 |  |  |  |
| CBLB | 1.20 | 0.00 | CXCL11 | 4.39 | 0.00 | IL12A | 2.25 | 0.00 | MELK | 1.34 | 0.00 | SRGN | 0.00 | -1.99 |  |  |  |
| CCL17 | 2.22 | 0.00 | CXCL13 | 2.77 | 0.00 | IL15 | 2.67 | 0.00 | MKI67 | 1.78 | 0.00 | SSX2 | 2.78 | 0.00 |  |  |  |
| CCL2 | 2.06 | 3.07 | CXCL8 | -1.70 | -2.34 | IL1B | -1.64 | -2.93 | MLANA | 2.60 | 0.00 | STAT1 | 1.64 | 0.00 |  |  |  |
| CCL20 | 2.87 | 0.00 | CXCL9 | 2.83 | 2.21 | IL22 | 2.04 | 0.00 | MMP2 | 3.21 | 0.00 | STAT4 | 2.12 | 0.00 |  |  |  |
| CCL21 | 2.08 | 0.00 | CXCR2 | 0.00 | -3.26 | IL2RA | 1.31 | 0.00 | MPO | 4.03 | 0.00 | STAT6 | 0.00 | -1.23 |  |  |  |
| CCL5 | -2.32 | 0.00 | CXCR6 | 1.86 | 0.00 | IL2RG | 0.00 | -1.64 | MRC1 | 0.00 | 2.08 | TAGAP | -1.66 | -2.54 |  |  |  |
| CCR4 | 1.90 | 0.00 | CYBB | -1.59 | 0.00 | IL4 | 2.42 | 0.00 | MS4A1 | 0.00 | -2.05 | TAP1 | -4.28 | 0.00 |  |  |  |
| CCR6 | 2.04 | 0.00 | DGAT2 | 2.63 | 0.00 | IL6 | 2.92 | 0.00 | MTOR | 2.84 | 0.00 | TBP | 1.32 | 0.00 |  |  |  |
| CD163 | 0.00 | 2.04 | DMBT1 | 3.29 | 0.00 | IL7 | 2.47 | 0.00 | NCAM1 | 1.96 | 0.00 | TBX21 | 3.83 | 0.00 |  |  |  |
| CD19 | 2.04 | 0.00 | EFNA4 | 4.16 | 0.00 | IRF4 | 2.07 | 0.00 | NECTIN2 | 1.86 | 0.00 | TIGIT | 1.78 | 0.00 |  |  |  |
| CD1D | 3.40 | 0.00 | EGFR | 1.89 | 0.00 | IRF9 | -1.14 | 0.00 | NFATC1 | 3.28 | 0.00 | TLR3 | 1.94 | 0.00 |  |  |  |
| CD22 | 2.89 | 0.00 | EIF2AK2 | -1.13 | 0.00 | IRS1 | 2.64 | 0.00 | NFKBIA | 2.46 | 0.00 | TLR7 | 1.37 | 0.00 |  |  |  |
| CD226 | 1.16 | 0.00 | ENTPD1 | 0.00 | -1.18 | ISG15 | 1.61 | 0.00 | NKG7 | -2.04 | 0.00 | TLR8 | -2.73 | -2.75 |  |  |  |
| CD247 | 1.42 | 0.00 | FAS | 0.00 | -1.26 | ISG20 | 2.79 | 0.00 | NOS2 | 3.09 | 0.00 | TNFRSF18 | 4.74 | 0.00 |  |  |  |
| CD33 | 2.42 | 0.00 | FCGR3B | -1.78 | -3.68 | ITGA1 | 1.77 | 2.03 | NRP1 | 3.01 | 2.11 | TNFRSF4 | 2.32 | 0.00 |  |  |  |
| CD38 | 1.49 | 0.00 | FOXM1 | 2.60 | 0.00 | ITGAE | 2.57 | 0.00 | NTN3 | 2.17 | 0.00 | TOP2A | 1.68 | 1.89 |  |  |  |
| CD40LG | 1.65 | 0.00 | FOXP3 | 1.69 | 0.00 | ITGAM | 1.45 | 0.00 | OAS1 | 1.40 | 0.00 | TNFRSF9 | 2.04 | 0.00 |  |  |  |
| CD44 | -1.68 | 0.00 | FUT4 | 2.05 | 0.00 | ITGAX | -1.96 | -3.35 | PECAM1 | 0.00 | -1.51 | TNFSF10 | -2.34 | 0.00 |  |  |  |
| CD52 | 2.27 | 0.00 | FYB | 0.00 | -2.19 | ITGB2 | 0.00 | -1.58 | PGF | 1.63 | 0.00 | TNFSF13B | -1.77 | -1.92 |  |  |  |
| CD53 | -1.39 | -1.57 | GATA3 | 2.53 | 2.40 | ITGB7 | 1.82 | 0.00 | PIK3CA | -1.18 | 0.00 | TNFSF14 | 0.00 | -1.77 |  |  |  |
| CD6 | 2.19 | 0.00 | GUSB | 1.76 | 1.66 | JAML | -1.20 | -2.37 | PIK3CD | 0.00 | -1.65 | TNFSF18 | 2.68 | 0.00 |  |  |  |

**Supplementary table S5:** 65 genes with SNVs found in both CTCs and corresponding metastatic samples (all SNVs are listed in the attached excel file).

| 1 "ABCB6" | 34 "MUC3A" |
| --- | --- |
| 2 "AC011343.1" | 35 "MUC5B" |
| 3 "AHNAK" | 36 "NCOR1" |
| 4 "AKAP13" | 37 "NEAT1" |
| 5 "ALMS1" | 38 "OFD1" |
| 6 "ANKRD12" | 39 "PCF11" |
| 7 "ANKRD50" | 40 "PCNT" |
| 8 "ANXA6" | 41 "PCNX1" |
| 9 "ARID1A" | 42 "PDPR" |
| 10 "BPTF" | 43 "PKHD1L1" |
| 11 "CCAR1" | 44 "PRMT2" |
| 12 "CCDC88C" | 45 "QSER1" |
| 13 "CERS5" | 46 "RBPJ" |
| 14 "CMPK2" | 47 "RNF213" |
| 15 "DOK5" | 48 "RP11-399K21.14" |
| 16 "FMN1" | 49 "SAP130" |
| 17 "FUS" | 50 "SHKBP1" |
| 18 "G3BP1" | 51 "SLC12A6" |
| 19 "GLB1" | 52 "SMC2-AS1" |
| 20 "GS1-124K5.11" | 53 "SPTAN1" |
| 21 "GTPBP1" | 54 "SSH2" |
| 22 "HERC1" | 55 "STIM1" |
| 23 "HIVEP1" | 56 "TARDBP" |
| 24 "HIVEP2" | 57 "TEP1" |
| 25 "IGF1R" | 58 "TET2" |
| 26 "JMJD1C" | 59 "TMEM131" |
| 27 "LAS1L" | 60 "TMOD2" |
| 28 "LINC01410" | 61 "TNIK" |
| 29 "LMOD1" | 62 "TRAPPC8" |
| 30 "LRRK2" | 63 "VPS13D" |
| 31 "MAPK1IP1L" | 64 "ZMAT2" |
| 32 "MDN1" | 65 "ZNF638" |
| 33 "MIR568" |  |

**Supplementary table S6:** All genes with SNVs and all SNVs found in Stage IV BC RNA-Seq. dataset which are also present in the COSMIC database (attached excel file).

**Supplementary table S7:** Results of SNV (n=10) validation using Sanger sequencing

| **RNA-Seq results** | | | | | | **Sanger sequencing results** | |
| --- | --- | --- | --- | --- | --- | --- | --- |
| **Samples** | **Genes** | **Mutated region** | **Reference. Allele** | **Mutated allele** | **Mutation** |  |  |
| 101738_CTC | *JAK1* | chr1: 64,855,599 | C | A | Stop-gain | Confirmed |  |
| 79388_CTC | *TRIM25* | chr17: 56,901,561 | TCG | T | Frameshift | Confirmed |  |
| 79388_Met | *DHRS9* | chr2: 169,083,565 | GA | G | Frameshift | Confirmed |  |
| 113488_CTC | *CDK12* | chr17: 39,471,212 | TG | T | Frameshift | Not confirmed |  |
| 113488_CTC | *MUC5B* | chr11: 1,245,888 | CCG | C | Frameshift | Confirmed |  |
| 113166_Met | *JAK2* | chr9: 5,066,709 | GC | G | Frameshift | Not confirmed |  |
| 113059_CTC | *LRBA* | chr4: 150,828,525 | TC | T | Frameshift | Not confirmed |  |
| 113488_CTC | *ROR1* | chr1: 64,049,744 | C | T | Stop-gain | Confirmed |  |
| 101738_CTC | *ZMIZ1* | chr10: 79,298,435 | C | A | Stop-gain | Confirmed |  |
| 113488_CTC | *IL31RA* | chr5: 55,910,630 | G | T | Stop-gain | Not confirmed |  |

**Supplementary table S8A:** Mutated driver genes (n=20) identified via RNA-Seq of MBC CTC and metastasis samples by gene

| Hugo_Symbol | Frame_Shift_Del | Frame_Shift_Ins | In_Frame_Del | Missense_Mutation | Nonsense_Mutation | Nonstop_Mutation | Splice_Site | total | Mutated_Samples |
| --- | --- | --- | --- | --- | --- | --- | --- | --- | --- |
| MUC16 | 3 | 0 | 0 | 20 | 0 | 0 | 0 | 23 | 7 |
| ARID1A | 0 | 0 | 0 | 6 | 0 | 0 | 0 | 6 | 5 |
| ANKRD12 | 0 | 0 | 0 | 5 | 1 | 0 | 0 | 6 | 4 |
| F5 | 0 | 0 | 0 | 5 | 0 | 0 | 0 | 5 | 4 |
| HIVEP1 | 0 | 0 | 0 | 5 | 0 | 0 | 0 | 5 | 4 |
| MACF1 | 1 | 0 | 0 | 4 | 0 | 0 | 0 | 5 | 4 |
| TET2 | 0 | 0 | 0 | 5 | 0 | 0 | 0 | 5 | 4 |
| ALMS1 | 0 | 0 | 0 | 3 | 1 | 0 | 0 | 4 | 4 |
| MKI67 | 0 | 0 | 0 | 4 | 0 | 0 | 0 | 4 | 4 |
| MUC3A | 0 | 0 | 0 | 4 | 0 | 0 | 0 | 4 | 4 |
| MUC12 | 0 | 0 | 0 | 5 | 0 | 0 | 0 | 5 | 3 |
| WIPF1 | 0 | 0 | 0 | 5 | 0 | 0 | 0 | 5 | 3 |
| AHNAK | 1 | 0 | 0 | 3 | 0 | 0 | 0 | 4 | 3 |
| BPTF | 1 | 0 | 0 | 2 | 1 | 0 | 0 | 4 | 3 |
| MDN1 | 0 | 0 | 0 | 4 | 0 | 0 | 0 | 4 | 3 |
| SOS1 | 0 | 0 | 0 | 4 | 0 | 0 | 0 | 4 | 3 |
| ZFHX4 | 0 | 0 | 0 | 4 | 0 | 0 | 0 | 4 | 3 |
| ARHGAP35 | 0 | 0 | 0 | 3 | 0 | 0 | 0 | 3 | 3 |
| BEST1 | 1 | 0 | 0 | 2 | 0 | 0 | 0 | 3 | 3 |
| CALM2 | 0 | 0 | 0 | 3 | 0 | 0 | 0 | 3 | 3 |

**Supplementary table S8B:** Mutated driver genes (n=20) identified via RNA-Seq of MBC CTC and metastasis samples by sample

| Sample | Frame_Shift_Del | Frame_Shift_Ins | In_Frame_Del | Missense_Mutation | Nonsense_Mutation | Nonstop_Mutation | Splice_Site | total |
| --- | --- | --- | --- | --- | --- | --- | --- | --- |
| 113488_CTC-PB | 30 | 1 | 4 | 652 | 46 | 1 | 15 | 749 |
| 101738_CTC_FollowUp-PB_FollowUp | 14 | 2 | 0 | 201 | 18 | 1 | 1 | 237 |
| 19065_CTC_FollowUp-PB_FollowUp | 9 | 0 | 0 | 84 | 5 | 1 | 1 | 100 |
| 79388_MET1-PB | 4 | 0 | 0 | 83 | 5 | 0 | 0 | 92 |
| 113457_CTC-PB | 4 | 2 | 0 | 51 | 3 | 0 | 1 | 61 |
| 19065_CTC-PB | 1 | 0 | 1 | 52 | 2 | 0 | 0 | 56 |
| 113059_CTC-PB | 7 | 0 | 0 | 46 | 2 | 0 | 0 | 55 |
| 36541_CTC_FollowUp-PB_FollowUp | 3 | 0 | 0 | 46 | 2 | 0 | 0 | 51 |
| 79556_MET1-PB | 1 | 0 | 0 | 41 | 3 | 0 | 0 | 45 |
| 81103_CTC-PB | 2 | 0 | 0 | 31 | 5 | 0 | 0 | 38 |
| 79412_2nd_CTC-PB | 4 | 0 | 0 | 33 | 0 | 0 | 0 | 37 |
| 68185_CTC-PB | 1 | 0 | 0 | 34 | 1 | 0 | 0 | 36 |
| 79388_CTC-PB | 1 | 0 | 0 | 34 | 0 | 0 | 0 | 35 |
| 112370_CTC-PB | 4 | 0 | 0 | 25 | 5 | 0 | 0 | 34 |
| 101738_CTC-PB | 1 | 0 | 0 | 23 | 2 | 1 | 0 | 27 |
| 36978_CTC_FollowUp-PB_FollowUp | 2 | 0 | 0 | 20 | 2 | 0 | 1 | 25 |
| 113166_CTC-PB | 0 | 0 | 0 | 21 | 1 | 0 | 0 | 22 |
| 112165_CTC-PB | 0 | 0 | 0 | 15 | 2 | 0 | 1 | 18 |
| 36978_CTC-PB | 2 | 0 | 0 | 15 | 1 | 0 | 0 | 18 |
| 36541_CTC-PB | 0 | 0 | 0 | 14 | 1 | 0 | 0 | 15 |
| 28089_MET1-PB | 1 | 0 | 0 | 10 | 0 | 0 | 0 | 11 |
| 113488_MET1-PB | 0 | 0 | 0 | 10 | 0 | 0 | 0 | 10 |
| 36978_MET2-PB | 1 | 0 | 0 | 6 | 2 | 0 | 0 | 9 |
| 68185_MET1-PB | 0 | 0 | 0 | 8 | 0 | 0 | 0 | 8 |
| 79412_MET1-PB | 0 | 0 | 0 | 7 | 0 | 0 | 0 | 7 |
| 81103_MET1-PB | 0 | 0 | 1 | 6 | 0 | 0 | 0 | 7 |
| 113166_MET1-PB | 1 | 0 | 0 | 5 | 0 | 0 | 0 | 6 |
| 112370_MET2-PB | 0 | 0 | 0 | 3 | 2 | 0 | 0 | 5 |
| 113457_MET1-PB | 0 | 0 | 0 | 5 | 0 | 0 | 0 | 5 |
| 36541_MET1-PB | 0 | 0 | 1 | 4 | 0 | 0 | 0 | 5 |
| 101738_MET1-PB | 0 | 0 | 0 | 4 | 0 | 0 | 0 | 4 |
| 113059_MET1-PB | 0 | 0 | 0 | 3 | 0 | 0 | 0 | 3 |
| 36978_MET1-PB | 0 | 0 | 0 | 3 | 0 | 0 | 0 | 3 |
| 28089_CTC-PB | 0 | 0 | 0 | 2 | 0 | 0 | 0 | 2 |
| 79412_2nd_MET-PB | 0 | 0 | 0 | 2 | 0 | 0 | 0 | 2 |
| 79412_CTC-PB | 0 | 0 | 0 | 2 | 0 | 0 | 0 | 2 |
| 112165_MET2-PB | 0 | 0 | 0 | 1 | 0 | 0 | 0 | 1 |
| 19065_MET2-PB | 0 | 0 | 0 | 1 | 0 | 0 | 0 | 1 |
| 79412_CTC_FollowUp-PB | 1 | 0 | 0 | 0 | 0 | 0 | 0 | 1 |
| 79556_CTC-PB | 0 | 0 | 0 | 1 | 0 | 0 | 0 | 1 |
| 79644_MET1-PB | 0 | 0 | 0 | 1 | 0 | 0 | 0 | 1 |

**Supplementary table S9:** Mutated IntOGen breast cancer driver genes in detected via RNA Seq of CTCs and metastasis samples

| Sample | Gene | ENSEMBLE ID | Chromosome | Position | Nucleotide | | Mutation type | Effect | |  | COSMIC ID |
| --- | --- | --- | --- | --- | --- | --- | --- | --- | --- | --- | --- |
| 113457_MET1-PB | PIK3CA | ENSG00000121879 | chr3 | 179234297 | A | G | missense_variant | MODERATE | tolerated(0.11) | protein_coding | 94986, 775 |
| 79388_CTC-PB | AKT1 | ENSG00000142208 | chr14 | 104772980 | A | G | missense_variant | MODERATE | deleterious(0) | protein_coding |  |
| 113488_CTC-PB | GATA3 | ENSG00000107485 | chr10 | 8058574 | G | T | missense_variant | MODERATE | deleterious(0.02) | protein_coding |  |
| 113488_CTC-PB | KMT2C | ENSG00000055609 | chr7 | 152145240 | A | T | missense_variant | MODERATE | deleterious(0) | protein_coding |  |
| 113488_CTC-PB | KMT2C | ENSG00000055609 | chr7 | 152176571 | G | A | missense_variant | MODERATE | deleterious_low_confidence(0.01) | protein_coding |  |
| 101738_CTC-PB | NCOR1 | ENSG00000141027 | chr17 | 16086434 | G | T | missense_variant | MODERATE | deleterious(0.03) | protein_coding |  |
| 79388_MET1-PB | NCOR1 | ENSG00000141027 | chr17 | 16070436 | G | T | synonymous_variant | LOW |  | protein_coding |  |
| 79556_MET1-PB | ATM | ENSG00000149311 | chr11 | 108281045 | A | G | synonymous_variant | LOW |  | protein_coding |  |
| 101738_MET1-PB | ARID1A | ENSG00000117713 | chr1 | 26731447 | C | A | missense_variant | MODERATE | deleterious_low_confidence(0) | protein_coding |  |
| 113166_CTC-PB | ARID1A | ENSG00000117713 | chr1 | 26779099 | A | G | missense_variant | MODERATE | deleterious(0) | protein_coding |  |
| 113488_CTC-PB | ARID1A | ENSG00000117713 | chr1 | 26731198 | G | T | missense_variant | MODERATE | deleterious_low_confidence(0) | protein_coding |  |
| 113488_CTC-PB | ARID1A | ENSG00000117713 | chr1 | 26780715 | C | G | missense_variant | MODERATE | tolerated(0.51) | protein_coding |  |
| 81103_MET1-PB | ARID1A | ENSG00000117713 | chr1 | 26771233 | T | C | missense_variant | MODERATE | deleterious(0) | protein_coding |  |
| 36978_CTC_FollowUp-PB_FollowUp | KMT2D | ENSG00000167548 | chr12 | 49031949 | G | C | synonymous_variant | LOW |  | protein_coding |  |
| 79412_2nd_CTC-PB | KMT2D | ENSG00000167548 | chr12 | 49028096 | T | C | missense_variant | MODERATE | deleterious(0.02) | protein_coding |  |
| 113488_CTC-PB | MYB | ENSG00000118513 | chr6 | 135190307 | G | T | missense_variant | MODERATE | deleterious(0) | protein_coding |  |
| 79412_2nd_CTC-PB | ZFP36L1 | ENSG00000185650 | chr14 | 68789679 | A | T | missense_variant | MODERATE | tolerated(0.07) | protein_coding |  |
| 113059_CTC-PB | BRCA2 | ENSG00000139618 | chr13 | 32338009 | C | A | synonymous_variant | LOW |  | protein_coding |  |
| 113488_CTC-PB | ANK3 | ENSG00000151150 | chr10 | 60076168 | A | G | synonymous_variant | LOW |  | protein_coding |  |
| 113488_CTC-PB | ANK3 | ENSG00000151150 | chr10 | 60172320 | G | T | synonymous_variant | LOW |  | protein_coding |  |
| 68185_CTC-PB | MYH9 | ENSG00000100345 | chr22 | 36295592 | T | C | missense_variant | MODERATE | tolerated(0.2) | protein_coding |  |
| 79412_2nd_CTC-PB | MYH9 | ENSG00000100345 | chr22 | 36294208 | C | T | missense_variant | MODERATE | tolerated(0.44) | protein_coding |  |
| 19065_CTC_FollowUp-PB | SPTAN1 | ENSG00000197694 | chr9 | 128613443 | G | A | synonymous_variant | LOW |  | protein_coding |  |
| 79556_MET1-PB | SPTAN1 | ENSG00000197694 | chr9 | 128585929 | C | T | synonymous_variant | LOW |  | protein_coding |  |
| 101738_CTC_FollowUp-PB_FollowUp | CCAR1 | ENSG00000060339 | chr10 | 68773064 | C | A | missense_variant | MODERATE | deleterious_low_confidence(0.02) | protein_coding |  |
| 19065_CTC_FollowUp-PB_FollowUp | CCAR1 | ENSG00000060339 | chr10 | 68753956 | C | A | stop_gained | HIGH |  | protein_coding |  |
| 79556_MET1-PB | CCAR1 | ENSG00000060339 | chr10 | 68755526 | T | A | missense_variant | MODERATE | deleterious(0) | protein_coding |  |
| 113059_CTC-PB | SETD2 | ENSG00000181555 | chr3 | 47057322 | A | G | synonymous_variant | LOW |  | protein_coding |  |
| 79388_MET1-PB | ARID2 | ENSG00000189079 | chr12 | 45852268 | C | G | missense_variant | MODERATE | deleterious_low_confidence(0) | protein_coding |  |
| 101738_CTC-PB | PBRM1 | ENSG00000163939 | chr3 | 52579078 | C | A | missense_variant | MODERATE | deleterious(0) | protein_coding |  |
| 113488_CTC-PB | MACF1 | ENSG00000127603 | chr1 | 39387751 | TC | T | frameshift_variant | HIGH |  | protein_coding |  |
| 19065_CTC-PB | MACF1 | ENSG00000127603 | chr1 | 39379359 | C | T | missense_variant | MODERATE | tolerated(0.14) | protein_coding |  |
| 101738_CTC_FollowUp-PB_FollowUp | AHNAK | ENSG00000124942 | chr11 | 62531285 | TTC | T | frameshift_variant | HIGH |  | protein_coding |  |
| 113457_CTC-PB | AHNAK | ENSG00000124942 | chr11 | 62526850 | A | G | missense_variant | MODERATE | tolerated(0.66) | protein_coding |  |
| 113457_CTC-PB | AHNAK | ENSG00000124942 | chr11 | 62526855 | G | T | missense_variant | MODERATE | deleterious(0) | protein_coding |  |
| 79412_2nd_CTC-PB | AHNAK | ENSG00000124942 | chr11 | 62528748 | C | T | missense_variant | MODERATE | tolerated(1) | protein_coding |  |
| 79412_MET1-PB | AHNAK | ENSG00000124942 | chr11 | 62523404 | G | A | synonymous_variant | LOW |  | protein_coding |  |
| 113488_CTC-PB | TAF1 | ENSG00000147133 | chrX | 71388350 | A | G | synonymous_variant | LOW |  | protein_coding |  |
| 36541_CTC_FollowUp-PB_FollowUp | TAF1 | ENSG00000147133 | chrX | 71368087 | A | G | missense_variant | MODERATE | deleterious(0) | protein_coding |  |
| 81103_CTC-PB | TAF1 | ENSG00000147133 | chrX | 71401539 | G | C | synonymous_variant | LOW |  | protein_coding |  |
| 81103_CTC-PB | TAF1 | ENSG00000147133 | chrX | 71460798 | C | T | synonymous_variant | LOW |  | protein_coding |  |
| 113457_CTC-PB | KMT2A | ENSG00000118058 | chr11 | 118504881 | T | C | missense_variant | MODERATE | deleterious_low_confidence(0) | protein_coding |  |
| 113488_CTC-PB | KMT2A | ENSG00000118058 | chr11 | 118502621 | C | T | synonymous_variant | LOW |  | protein_coding |  |
| 113488_CTC-PB | KMT2A | ENSG00000118058 | chr11 | 118505677 | C | T | missense_variant | MODERATE | deleterious_low_confidence(0) | protein_coding | 4514519, 4514518 |
| 101738_CTC_FollowUp-PB_FollowUp | MTOR | ENSG00000198793 | chr1 | 11228757 | A | T | missense_variant | MODERATE | tolerated(0.9) | protein_coding |  |
| 113488_CTC-PB | ASH1L | ENSG00000116539 | chr1 | 155341996 | G | A | synonymous_variant | LOW |  | protein_coding |  |
| 79412_2nd_CTC-PB | ASH1L | ENSG00000116539 | chr1 | 155347866 | A | T | synonymous_variant | LOW |  | protein_coding |  |
| 113488_CTC-PB | CDK12 | ENSG00000167258 | chr17 | 39471212 | TG | T | frameshift_variant | HIGH |  | protein_coding |  |
| 113488_CTC-PB | CDK12 | ENSG00000167258 | chr17 | 39471267 | G | A | missense_variant | MODERATE | tolerated_low_  confidence(0.08) | protein_coding |  |
| 113488_CTC-PB | KALRN | ENSG00000160145 | chr3 | 124430661 | G | A | synonymous_variant | LOW |  | protein_coding |  |
| 113488_CTC-PB | MGA | ENSG00000174197 | chr15 | 41766108 | C | A | missense_variant | MODERATE | tolerated(0.36) | protein_coding |  |
| 101738_CTC-PB | ARHGAP35 | ENSG00000160007 | chr19 | 46919370 | C | A | missense_variant | MODERATE | tolerated(0.08) | protein_coding | 712211, 712210 |
| 101738_CTC_FollowUp-PB_FollowUp | ARHGAP35 | ENSG00000160007 | chr19 | 46918760 | G | T | missense_variant | MODERATE | deleterious(0) | protein_coding |  |
| 36541_CTC-PB | ARHGAP35 | ENSG00000160007 | chr19 | 46921752 | T | C | missense_variant | MODERATE | tolerated(0.65) | protein_coding |  |
| 113488_CTC-PB | NUP98 | ENSG00000110713 | chr11 | 3760628 | T | C | splice_acceptor_  variant | HIGH |  | protein_coding |  |
| 19065_CTC_FollowUp-PB_FollowUp | NUP98 | ENSG00000110713 | chr11 | 3712722 | G | T | missense_variant | MODERATE | deleterious(0) | protein_coding |  |
| 36978_CTC_FollowUp-PB_FollowUp | SMARCA4 | ENSG00000127616 | chr19 | 11013076 | G | A | missense_variant | MODERATE | tolerated(0.06) | protein_coding | 1153286, 990746 |
| 79388_MET1-PB_ | BCOR | ENSG00000183337 | chrX | 40074537 | G | A | missense_variant | MODERATE | tolerated(0.06) | protein_coding |  |
| 101738_CTC-PB | BPTF | ENSG00000171634 | chr17 | 67945450 | C | T | stop_gained | HIGH |  | protein_coding |  |
| 101738_CTC_FollowUp-PB_FollowUp | BPTF | ENSG00000171634 | chr17 | 67854698 | C | A | missense_variant | MODERATE | deleterious(0) | protein_coding |  |
| 101738_CTC_FollowUp-PB_FollowUp | BPTF | ENSG00000171634 | chr17 | 67945650 | AC | A | frameshift_variant | HIGH |  | protein_coding |  |
| 113457_CTC-PB | BPTF | ENSG00000171634 | chr17 | 67854373 | A | G | synonymous_variant | LOW |  | protein_coding |  |
| 113488_CTC-PB | BPTF | ENSG00000171634 | chr17 | 67945965 | C | T | synonymous_variant | LOW |  | protein_coding |  |
| 28089_MET1-PB | BPTF | ENSG00000171634 | chr17 | 67853994 | A | G | missense_variant | MODERATE | deleterious(0) | protein_coding |  |
| 36541_CTC_FollowUp-PB_FollowUp | MED24 | ENSG00000008838 | chr17 | 40022714 | A | G | missense_variant | MODERATE | deleterious(0) | protein_coding |  |
| 68185_CTC-PB | CSDE1 | ENSG00000009307 | chr1 | 114739820 | A | T | missense_variant | MODERATE | tolerated(0.11) | protein_coding |  |
| 101738_CTC_FollowUp-PB_FollowUp | EP300 | ENSG00000100393 | chr22 | 41150126 | C | T | synonymous_variant | LOW |  | protein_coding |  |
| 113457_CTC-PB | EP300 | ENSG00000100393 | chr22 | 41176787 | T | A | stop_gained | HIGH |  | protein_coding |  |
| 101738_CTC_FollowUp-PB_FollowUp | PIK3CB | ENSG00000051382 | chr3 | 138691070 | G | T | missense_variant | MODERATE | deleterious(0) | protein_coding |  |
| 113488_CTC-PB | SOS1 | ENSG00000115904 | chr2 | 38986005 | G | A | missense_variant | MODERATE | tolerated_low_  confidence(0.47) | protein_coding |  |
| 36541_CTC_FollowUp-PB_FollowUp | SOS1 | ENSG00000115904 | chr2 | 38985981 | A | G | missense_variant | MODERATE | tolerated_low_  confidence(1) | protein_coding |  |
| 36541_CTC_FollowUp-PB_FollowUp | SOS1 | ENSG00000115904 | chr2 | 38986003 | A | G | missense_variant | MODERATE | tolerated_low_  confidence(0.21) | protein_coding |  |
| 68185_MET1-PB | NR4A2 | ENSG00000153234 | chr2 | 156328493 | T | C | missense_variant | MODERATE | tolerated(0.11) | protein_coding |  |
| 113488_CTC-PB | TNPO1 | ENSG00000083312 | chr5 | 72891886 | C | A | missense_variant | MODERATE | deleterious(0) | protein_coding |  |
| 113488_CTC-PB | FUS | ENSG00000089280 | chr16 | 31188336 | C | A | missense_variant | MODERATE | tolerated(0.22) | protein_coding |  |
| 113488_CTC-PB | FUS | ENSG00000089280 | chr16 | 31190811 | C | A | synonymous_variant | LOW |  | protein_coding |  |
| 79388_MET1-PB | FUS | ENSG00000089280 | chr16 | 31182580 | C | G | missense_variant | MODERATE | tolerated(0.07) | protein_coding |  |
| 113488_CTC-PB | CAST | ENSG00000153113 | chr5 | 96741289 | T | A | synonymous_variant | LOW |  | protein_coding |  |
| 113488_CTC-PB | RBM5 | ENSG00000003756 | chr3 | 50093744 | G | C | missense_variant | MODERATE | deleterious(0.02) | protein_coding |  |
| 113488_CTC-PB | STIP1 | ENSG00000168439 | chr11 | 64194509 | C | A | missense_variant | MODERATE | deleterious(0.01) | protein_coding |  |
| 36541_CTC_FollowUp-PB_FollowUp | TFDP1 | ENSG00000198176 | chr13 | 113635984 | C | G | missense_variant | MODERATE | deleterious(0.01) | protein_coding |  |
