# Supplementary material for: Circulating Tumor Cell Transcriptomics as Biopsy Surrogates in Metastatic Breast Cancer
Source: Ann Surg Oncol. 2022 Jan 9;29(5):2882–94. doi: 10.1245/s10434-021-11135-2 (PMC8989945; doi:10.1245/s10434-021-11135-2)
Supplement: Supplementary file 5 — Supplementary file5 (DOCX 26 kb) [file 10434_2021_11135_MOESM5_ESM.docx]

*Sample preparation*

Peripheral blood (200 μL) was harvested into 1 ml RNA later and processed using the RiboPure Kit from Ambion (Thermo Fisher Scientific). Total RNA was isolated from metastases using TRIzol (Thermo Fisher Scientific, Waltham, MA). Shearing of cDNA was performed using a Covaris S220 Ultrasonicator (Covaris, Woburn, MA). A NanoDrop 2000 (Thermo Fisher Scientific) and 2100 Bioanalyzer (Agilent Technologies, Santa Clara, CA) were used for quality control and concentration measurements.

*Sanger Sequencing*

Sanger sequencing primers were designed using Primer3Plus2 (supplementary table S1). The T100 Thermal Cycler Polymerase Chain Reaction (PCR) instrument (Bio-Rad, Hercules, CA, USA) was used for DNA amplification. Approximately 200-400 base pair sequences surrounding each mutation in CTCs and metastases was amplified using a PCRBIO Taq DNA Polymerase Kit (Genesee, El Cajon, CA, USA). PCR product size was confirmed with agarose gel electrophoresis. Purification of the amplified PCR product and

*RNA-Seq data Analysis*

FASTQ files read quality and adaptor content was assessed with FastQC [1] and reads were trimmed using Trimmomatic [2]. High-quality reads were mapped to the human genome (GRCh38.p7), and read counts were obtained using the Spliced Transcripts Alignment to a Reference (STAR ver. 2.5.2b) aligner [3]. Gene feature references were obtained from the GENCODE database. Normalized reads per kilobase million (RPKM) values were produced using the edgeR package [4]. Differential gene expression analysis was performed with the R/Bioconductor package DESeq2 [5], and p-values were corrected for multiple testing using the Benjamini and Hochberg method; p-values < 0.05 after correction were considered significant. The PCA was done using the function plotPCA from DESeq2. The input data for the plot was transformed read counts. Read counts were transformed using a Variance Stabilizing Transformation. Differential gene expression using PB samples was calculated to determine the degree of gene expression in CTCs and metastasis against the background of WBCs (here referred to as normalization against the PB background). The Oncomine Immune Response Research Assay (Thermo Fisher Scientific) gene list was used to query expression of immuno-oncology (IO) targets.

For somatic SNV calling, the FASTQ files were processed following the Best Practices Workflow for variant calling with RNA-Seq from the Broad Institute. The bam files were analyzed using two different methods and the intersect used as the final call set: 1) MuTect2 [6], which follows a probabilistic approach, and 2) VarScan 2 [7], which uses a heuristic algorithm with statistical thresholds for filtering. The final SNV set was annotated using the Variant Effect Predictor software. The FASTQ files, as well as the corresponding read count files for each sample were deposited in the Gene Expression Omnibus database (GSE113890). Visualization of SNVs was done via the RCircos package for R [8]. The COSMIC database was used to find known SNVs in our data set. The driver gene analysis was done using Maftools [9]. In addition, 184 known driver genes in BC from the Integrative Onco Genomics database (<http://www.intogen.org/mutations/>) [10] were investigated in our SNV dataset. Computational analyses were done on the University of Southern California’s Center for High-Performance Computing Cluster (<https://hpcc.usc.edu>).

We curated a list of 64 BC related genes with clinical and preclinical therapeutic, prognostic or diagnostic implications [11] (Supplementary table S2) representing breast cancer relevant pathways (EGFR/RAF/MEK, IGF-1/PI3K/AKT/mTOR, WNT/NOTCH/Hedgehog/FGF/MET, DNA damage repair, cell cycle, hormone receptor signaling, tumor suppressors, and tumor immunology).

**References**

1. Andrews S, K.F., Seconds-Pichon A, Biggins F, Wingett S, *FastQC. A quality control tool for high throughput sequence data.* Babraham Bioinformatics. Babraham Institute.

2. Bolger, A.M., M. Lohse, and B. Usadel, *Trimmomatic: a flexible trimmer for Illumina sequence data.* Bioinformatics, 2014. **30**(15): p. 2114-20.

3. Dobin, A., et al., *STAR: ultrafast universal RNA-seq aligner.* Bioinformatics, 2013. **29**(1): p. 15-21.

4. Robinson, M.D., D.J. McCarthy, and G.K. Smyth, *edgeR: a Bioconductor package for differential expression analysis of digital gene expression data.* Bioinformatics, 2010. **26**(1): p. 139-40.

5. Love, M.I., W. Huber, and S. Anders, *Moderated estimation of fold change and dispersion for RNA-seq data with DESeq2.* Genome Biol, 2014. **15**(12): p. 550.

6. Cibulskis, K., et al., *Sensitive detection of somatic point mutations in impure and heterogeneous cancer samples.* Nat Biotechnol, 2013. **31**(3): p. 213-9.

7. Koboldt, D.C., et al., *VarScan 2: somatic mutation and copy number alteration discovery in cancer by exome sequencing.* Genome Res, 2012. **22**(3): p. 568-76.

8. Zhang, H., P. Meltzer, and S. Davis, *RCircos: an R package for Circos 2D track plots.* BMC Bioinformatics, 2013. **14**: p. 244.

9. Mayakonda, A., et al., *Maftools: efficient and comprehensive analysis of somatic variants in cancer.* Genome Res, 2018. **28**(11): p. 1747-1756.

10. Gonzalez-Perez, A., et al., *IntOGen-mutations identifies cancer drivers across tumor types.* Nat Methods, 2013. **10**(11): p. 1081-2.

11. Kaur, P., et al., *Comparison of TCGA and GENIE genomic datasets for the detection of clinically actionable alterations in breast cancer.* Sci Rep, 2019. **9**(1): p. 1482.
